# Bridging the gap between health insurance eligibility and uptake: A multistate quantitative analysis of entitlement-based verification patterns under India’s PM-JAY Scheme

**DOI:** 10.1371/journal.pgph.0007451

**Published:** 2026-10-13

**Authors:** Swati Srivastava, Tads Ciecierski-Holmes, Christoph Strupat, Divya Parmar, Nishant Jain, Manuela De Allegri

**Affiliations:** 1 Heidelberg Institute of Global Health, Medical Faculty and University Hospital, Heidelberg University, Heidelberg, Germany; 2 University of Cambridge, School of Clinical Medicine, Addenbrooke’s Hospital, Cambridge, United Kingdom; 3 Economic and Social Systems, German Institute of Development and Sustainability (IDOS), Bonn, Germany; 4 Department of Population Health Sciences, School of Life Course and Population Sciences, King’s College London, King’s College London, London, United Kingdom; 5 Asian Development Bank, Mandaluyong City, Metro Manila, Philippines; Marie Adelaide Leprosy Center, PAKISTAN

## Abstract

Publicly-funded health insurance schemes, wherein governments subsidize insurance contributions for disadvantaged groups, are a key strategy under universal health coverage. India’s Pradhan Mantri Jan Arogya Yojana (PM-JAY) uses an entitlement-based approach whereby approximately 700 million beneficiaries are automatically eligible for scheme benefits, subject to a simplified identity verification procedure instead of enrolment. This study investigates the determinants of undergoing verification among PM-JAY’s target population to identify underserved groups and inform strategies to expand coverage. We used cross-sectional survey data from 2019-2020, covering 50,002 individuals across five states. We examined individual and household characteristics associated with verification using logistic regression models with district-level random effects, and beneficiary experiences with the verification process. About one-fifth of eligible individuals had verified their status within a year of launch. Verification was more likely among older adults, with those ≥60years having higher odds than those 0–15 years of age (adjusted Odd Ratio [AOR] 2.339; 95% CI 2.071-2.641). Those with higher education had higher odds than those with no formal education (AOR 2.303; 95% CI 2.048-2.590), those with prior PFHI enrolment had higher odds compared to those not enrolled (AOR 1.947; 95% CI 1.827-2.075), and those with chronic illness had higher odds than those without (AOR 1.175; 95% CI 1.071-1.289). Women and household members other than spouses were less likely to verify. Disadvantaged castes and minority religions demonstrated relatively higher verification rates. District-level factors explained nearly one-third of variability, indicating that infrastructure and implementation capacity differences influence uptake. Many beneficiaries reported long wait times, varying documentation demands, and informal payments during verification. Addressing these barriers requires gender-sensitive outreach, streamlined household-wide verification, and strengthened local health system capacity. Policy reforms that simplify verification, ensure equitable access, and strengthen health infrastructure are vital to advancing effective universal health coverage for India’s most vulnerable populations.

## Introduction

Publicly-funded health insurance (PFHI), in which governments subsidize health insurance contributions for disadvantaged groups, is a key strategy in low- and middle-income countries (LMIC) to advance towards universal health coverage (UHC). PFHI reduce financial barriers and increase utilization of health services, especially among poor and underserved populations, although evidence of PHFI’s effects on health outcomes remains inconclusive [1]. Beneficiary participation in PHFI is shaped by the scheme’s design, implementation, and public perceptions about these aspects; greater participation is surmised to lead to greater service utilization and a reduction in financial access barriers [1].

Formal identification and opt-in procedures are the gateway to beneficiary participation in health insurance schemes. Traditionally, social health insurance schemes have used mandatory enrolment procedures comprising identity confirmation and payroll- or time-bound premium contributions, both of which are challenging in LMICs. Identification of targeted beneficiaries is difficult due to inadequate data and cumbersome administrative procedures. Payroll contributions are not a reliable revenue source due to large informal economies, and serve as a widespread financial barrier among the poorest and most marginalized, deterring participation [2]. PFHI scheme designs seek to address shortcomings of beneficiary contributions by having governments partly or wholly subsidize payments for selected groups, through tax revenues, cross-subsidization across beneficiaries, and/or other government transfers and subsidies. Many PHFI schemes thus adopt tiered enrolment contributions based on ability to pay, or other means of linking contributions to socio-economic status. However, evidence show that periodic renewal requirements [3], co-payments [4] and cumbersome enrolment procedures impede participation [5] in such schemes. As a result, PHFI enrolment has been consistently low across LMICs [6]. A few schemes, such as Indonesia’s JKN and Ghana’s NHIS, have simplified traditional enrolment processes, often through mobile App-based solutions, and exempted premium contributions for some of the poorest and most marginalized beneficiaries. Despite these measures, enrolment in these schemes remains low, especially among high-priority exemption groups [7–9]. The Philippines PhilHealth program went one step further by introducing an immediate eligibility policy in 2019, under which entitlement to benefits was decoupled with premium payments and entails only a **verification** (or identity confirming) procedure [10]. Although verification aimed to overcome barriers to traditional enrolment procedures alongside the removal of premium contributions for select groups, it remains an additional step that may discourage uptake. Preliminary evidence from one study on PhilHealth using administrative and qualitative data shows that while membership and utilization have both increased, challenges such as the need for identification numbers, additional documentation, and facility-level capacity issues with implementing manual verification procedures limit participation, especially for the most vulnerable beneficiaries [10]. There is a considerable evidence gap in understanding who is still missing out on PFHI coverage despite entitlement-based eligibility through verification procedures decoupled from beneficiary contributions.

In this study, we examine eligibility verification under the **Pradhan Mantri Jan Arogya Yojana** (PM-JAY) PFHI scheme in India, which provides inpatient coverage of up to INR 500,000 (EUR 5000 in 2025) annually per household for inpatient services [11]. PM-JAY was formed by consolidating the previous national PHFI, the national Rashtriya Swasthya Bima Yojana (RSBY) scheme and other, state-level PFHI schemes. Historically, many Indian states started launching PFHI schemes after the passage of the Insurance Regulatory and Development Authority of India Act in 1999 which enabled health insurance reform and entry of private insurance providers; these schemes covered inpatient services and included the 2007 Rajiv Aarogyasri Scheme (in Andhra Pradesh), the 2008 central RSBY, and the 2009 Vajpayee Arogyasree Scheme, among others [12]. RSBY followed these schemes, and was the largest such scheme for informal workers and the marginalized sections of society, but suffered from cumbersome enrolment procedures [13]. Under all of these schemes and under RSBY, beneficiaries had to make a nominal contribution of INR 30 per household at the time of enrolment for scheme administrative expenses; this was paid annually for membership renewal. Additionally, enrolment was only possible once a year during enrolment drives; if beneficiaries missed these they would have to wait until the next year. PM-JAY has no contribution requirements. Further, PM-JAY is unique because like PhilHealth, it simplified and eliminated bureaucratic enrolment procedures by moving to an entitlement-based eligibility: nearly 500 million poor and vulnerable individuals identified in the 2011 Socio-Economic Caste Census (SECC) are automatically eligible, subject to verification of their identity, which (unlike RSBY) can also take place at the time of hospital admission. Indian states have since expanded eligibility criteria, adding another 200 million eligible beneficiaries, making PM-JAY one of the largest PFHI schemes globally with nearly 700 million potential enrolees [11,14]. PM-JAY is therefore a central pillar of India’s progress towards UHC, with an explicit focus on extending financial protection and access to hospital care to socioeconomically vulnerable households, consistent with the Sustainable Development Goal commitment to “leave no one behind” [15].

Despite simplifying the eligibility process and eliminating contributions, verification numbers remain low: by 2021, only 162 million PM-JAY cards were issued for the 700 million potential enrolees [14]. This suggests that either people do not verify eligibility because of the provision of being able to perform it before hospital admission, or because of persistent barriers in transitioning eligibility into verified participation. We examine who is more likely (or not) to undergo verification among PM-JAY’s target beneficiaries, to identify groups for outreach and to inform strategies to enhance coverage.

## Methods

### Ethics statement

We obtained ethical approval from the Ethics Commission of the Medical Faculty, Heidelberg University, Germany via approval number 2019-05-09 ZB 25519, and from Sigma Institutional Review Board, New Delhi, India via approval number 10 057/IRB/19-20. All respondents provided written (or when unable, with thumb-print) informed consent to participate in the study. No minors were included in the study as respondents, as all responses were from adult household members. The research study adhered to the Declaration of Helsinki.

### The PM-JAY verification process

Eligibility verification is conducted through kiosks at community centers, public health facilities, private empanelled hospitals, Common Service Centers (service centers set up within municipalities or villages to oversee multiple government programs) telephone helpline or via the online “Am I eligible” online portal. Beneficiary data are validated against an electronic Beneficiary Identification System (BIS) using government IDs such as an Aadhaar Card, ration cards, mobile numbers, or official invitation letters to join [16]. This process is performed for every member of a household, and can be performed at any time, even preceding hospital admission. Once a beneficiary has been identified, their information is sent to a state appointed insurance company, state health agency, or contracted agency, for approval, and they are provided with a ‘Gold Card’ (a paper or smart card indicating PM-JAY eligibility) as proof of verification.

### Study design

We performed a cross-sectional analysis of household data collected from PM-JAY eligible households.

### Sampling and data collection

**Sampling method and procedures:** We used a subset of household survey data collected for a demand- and supply-side evaluation of the PM-JAY [17]. Data were collected from PM-JAY-eligible households in 5 Indian States (Bihar, Chhattisgarh, Gujarat, Meghalaya, and Uttar Pradesh) during December 2019-February 2020; this period constituted the early implementation stage of PM-JAY. The states were selected in consultation with policy stakeholders to ensure geographic diversity and to represent different models of PM-JAY implementation. In this paper, we did not use data from two states in the original study (Kerala and Tamil Nadu) because they have different verification procedures compared to national guidelines.

Within each state, two districts (three in Bihar and Uttar Pradesh) classified as well- or average-performing in PM-JAY implementation were chosen; low-performing districts were excluded due to incomplete scheme implementation and expected poor outcomes. In each district, the primary sampling units (PSUs) comprised villages in rural or peri-urban areas (<7,500 inhabitants). Fifty PSUs were randomly selected to be representative of district-level PM-JAY characteristics, including the number of eligible households, number of claims, and broader demographic features (e.g., age, sex, marital status, and household composition) [18]. From each PSU, 15 households were randomly selected. This entailed a sample of approximately 1150 households per district. Further details of the sampling strategy are provided in De Allegri et al. (2020) [17]. The districts have been anonymized for analysis.

**Tool development and validation:** The survey questionnaire captured household characteristics, socioeconomic background, and PM-JAY-specific information, including whether members had undergone verification and their experience of this process. A survey module on household consumption expenditure as a proxy for household wealth was developed, using a 30-day recall period for more frequently consumed items and a 365-day recall period for large purchases. Survey questions were developed using standard questions used in other validated surveys, such as the Indian government’s Survey on Household Social Consumption: Health [19], the Household Consumption Expenditure Survey [19] and the Demographic and Health Surveys. Survey questions on PM-JAY-specific information were developed using PM-JAY scheme beneficiary process guidelines [20]. The questionnaire was translated into six regional languages. The translated questionnaire was piloted in peri-urban areas of Ahmedabad (Gujarat state) in June 2019, in Ghaziabad (Uttar Pradesh state) in July 2019, and in Nadia (West Bengal state) in August 2019. The piloting exercise was used to test the applicability, flow, consistency and language of questions, skip patterns, and estimate the length of interviews. Special care was taken to pilot questions on consumption expenditure of the household and their recall periods. Data were collected by locally recruited interviewer teams from the study districts who were fluent in the local language, using computer-assisted personal interviewing (CAPI) on digital devices. More detailed information is available in the study protocol [17].

**Respondent selection process:** The head of household, or another knowledgeable adult in the sampled household served as the questionnaire respondent.

**Quality assurance measures:** Quality assurance measures before data collection included use of validated questions, piloting of the questionnaire, development of a survey field manual, and two week-long training of interviewers. During data collection, built-in electronic checks in the CAPI, monitoring of data collection and daily debrief sessions. After data collection, detailed data dictionaries and documentation of cleaning decisions were used to maintain data quality.

21 households corresponding to 108 individuals from the original dataset were dropped from the analysis due to incomplete household consumption data or missing age of individual household members. 3 individuals were excluded as they identified as transgender, so we did not have a sufficient sample to comment on this gender group. 55 individuals were removed for having an unknown age. A further 147 were excluded as they responded that they were enrolled in a government health insurance scheme, which should have disqualified them for PM-JAY. As the number of missing households was small (0.4% of all households in the original dataset), and the number of excluded individuals in total was small (313, 0.6% of all individuals) we did not perform any imputation for missing values. The final dataset included 9,833 households (50,002 individuals). From these households, the 2,276 household heads (or another available adult member) who knew about PM-JAY, were eligible for the program, and had undergone the verification process were asked detailed questions about their experiences. We received all data as fully anonymized, secondary data.

### Variables and measurement

The primary outcome variable was whether an individual had verified their eligibility for PM-JAY, hereafter referred to as completing the PM-JAY verification procedure. Explanatory variables included both household and individual level variables (Table 1).

**Table 1:** Descriptive statistics of variables included in the analysis (N = 50,002).

Variable	Definition	%	N	Expected sign of association with outcome variable
Outcome Variable				
Individual has completed verification	0 = No	78	39,294	NA
	1 = Yes	22	11,018	
Explanatory Variables				
*Individual Level Variables*				
Relationship to Head of Household	1 = Household Head	20	9,804	+/-
	2 = Spouse	15	7,519	
	3 = Other	65	32, 679	
Sex	0 = Male	50	25,330	+/-
	1 = Female	50	24,982	
Age	0 = 0–15 years	33	16,637	+/-
	1 = 16–29 years	26	12,785	
	2 = 30–39 years	12	6,051	
	3 = 40–49 years	11	5,651	
	4 = 50–59 years	8	3,839	
	5 = 60 + years	10	5,039	
Marital Status	1 = Married	44	22,123	- (not married)
	2 = Not Married	56	27,879	
Education	0 = No formal education	38	18,859	- (for less education) + (for more educated)
	1 = Up to Primary Level	25	12,666	
	2 = Primary to Secondary Level	32	16,150	
	3 = Above Secondary Level	5	2,327	
Hospitalisation	0 = Not hospitalised in the past 365 days	96	48,243	+ (for those who have been hospitalised)
	1 = Has been hospitalised in the past 365 days	4	1,759	
Chronic Illness	0 = Had no illness lasting more than 3 months	92	46,239	+ (for those with chronic illness)
	1 = Has had an illness lasting more than 3 months	8	3,753	
*Household Level Variables*				
Household Size	0 = Five or fewer household members	44	21,741	- (larger households)
	1 = More than five household members	56	28,261	
Caste	1 = Scheduled Caste	32	16,016	+/-
	2 = Scheduled Tribe	30	15,066	
	3 = Other Backwards Class	32	15,694	
	4 = General/Other or No Caste	6	2,956	
Religion	1 = Hindu	80	39,921	+/-
	2 = Others	20	10,081	
Other Publicly Funded Health Insurance	0 = Not previously enrolled in a PFHI	50	25,115	+ (if previously enrolled)
	1 = Previously enrolled in a PFHI	50	24,887	
Yearly Household Consumption Expenditure Quintiles	1^st^ Quintile - Lowest	20	9,996	+ (for higher income individuals)
	2^nd^ Quintile	20	10,000	
	3^rd^ Quintile	20	9,990	
	4^th^ Quintile	20	10,005	
	5^th^ Quintile - Highest	20	10,011	
*District*	1 = District A, Bihar	11	5,335	Not applicable
	2 = District B, Bihar	7	3,750	
	3 = District C, Bihar	10	4,788	
	4 = District D, Chhattisgarh	7	3,476	
	5 = District E, Chhattisgarh	6	2,849	
	6 = District F, Gujarat	8	4,013	
	7 = District G, Gujarat	8	4,016	
	8 = District H, Meghalaya	7	3,555	
	9 = District I, Meghalaya	8	4,219	
	10 = District J, Uttar Pradesh	8	4,169	
	11 = District K, Uttar Pradesh	9	4,717	
	12 = District L, Uttar Pradesh	10	5,115	

Household-level variables were household size, estimated household consumption expenditure (as a proxy for income), caste, and religion. Household consumption expenditure was estimated from detailed item-wise reports of the quantity and value of goods and services consumed over specified reference periods (for example, the last 30, or 365 days, depending on the item group). For each household, expenditures across all reported items were summed to obtain total household consumption expenditure over the reference period, which was then converted to a monthly value and divided by household size to derive monthly per capita consumption expenditure (MPCE). Within each state, households were divided into quintiles of MPCE to account for state-level variations in consumption and wealth. This estimation followed the exact method and item lists used in the Indian national Household Social Consumption Surveys [19].

Individual-level variables included relationship to household head, sex, age (grouped into categories reflecting key life-course and labour-market stages [0–15, 16–29, 30–39, 40–49, 50–59, and 60 + years], consistent with commonly used demographic age classifications and with the distinction between dependent, prime working-age, and older adult populations), marital status, education, chronic illness in the last 3 months, hospitalization in the past year, and coverage by a previous state PFHI. In the absence of dedicated literature on verification uptake in the context of PFHI, we drew our hypotheses based on the literature addressing barriers and facilitators on insurance uptake. We hypothesized economic disadvantage (low income, low education, marginalized caste or religion) to reduce verification. Household structure could also influence outcomes: those in larger households or distantly related to the household head may lack access to necessary information or identification documents. We expected prior enrolment in a state PFHI or membership in a targeted end-beneficiary group to facilitate verification. We anticipated that poorer health status, particularly among the elderly or those with chronic illness, to increase verification due to higher service needs.

We also explored factors related to the different aspects of the verification process. For example, household heads were asked about the place they verified their enrolment, how long they had to wait before the procedure was initiated, how many documents were required, whether they paid for verification or to receive a Gold Card, and how satisfied they were with the verification process. This analysis provided insights into both procedural barriers and user experience of the PM-JAY verification process.

### Analytical approach

We used bivariate analysis using chi-squared tests to examine whether individuals who had completed the verification process and those that had not were different. These bivariate analyses were descriptive and intended to summarize the distribution of key covariates by verification status and to identify patterns for presentation in Table 1; they were not used for variable selection in the multivariable model. We used a logistic regression analysis to examine variables that were associated with completing PM-JAY verification. The logistic regression model estimated coefficients for individual and household level variables, and uses random effects at the district level, attributing remaining variance in the model to the district level effects. We used district random effects to account for clustering of households within districts, aligned with the strategy of sampling PSUs to be representative at the district level. Further, as the period of data collection was under the early implementation stage of PM-JAY and many implementation processes (including awareness generation campaigns and access to verification centers) were not standardized across districts, we hypothesized that district-specific contextual characteristics may have affected verification.

As individuals are nested within households, which in turn are nested within districts, we adjusted the standard errors of the regression model estimates to account for household level clustering using a nonparametric cluster bootstrap run 500 times. We resampled with replacement from the observed data at the household level, refit the model on each resampled dataset to obtain a distribution of coefficient estimates, and constructed 95% confidence intervals for each fixed-effect coefficient using the percentile method.

We did not add a household-level random effect because our substantive focus is on individual- and household-level predictors rather than household-specific latent effects, we therefore treat the district random effect as the primary clustering adjustment and acknowledge the possibility of residual within-household correlation as a limitation.

All covariates included in the multivariable model were specified a priori based on conceptual frameworks of health insurance uptake and our hypotheses described above. The model included the following covariates: household size; MPCE quintile; caste; religion; relationship to household head; sex; age group; marital status; education; chronic illness in the last 3 months; hospitalization in the past year; and prior coverage by a state PFHI. These variables were retained regardless of their statistical significance in bivariate or univariable analyses to avoid selection bias and to ensure adjustment for key confounders identified in the literature.

The estimated model is defined below. All analyses were performed using R Studio v.2022.12.0.


$$\begin{array}{c} \text{logit}\left(E\left[Y_{ijk}|b_{i}\right]\right)=b_{i}+\beta_{\text{0}}+\beta_{\text{1}}X_{ijk}+\beta_{\text{2}}W_{ik} \end{array}$$

$$\begin{array}{c} Var\left(E\left[Y_{ijk}|b_{i}\right]\right)=E\left[Y_{ijk}|b_{i}\right](\text{1}-E\left[Y_{ijk}|b_{i}\right]) \end{array}$$

In a separate analysis and only for the relevant sub-sample, descriptive statistics were used to explore the experiences of household heads who had undergone the verification process.

Reporting of all items in this study adhered to the STROBE statement for reports of cross-sectional studies.

## Results

Table 1 provides summary statistics of the survey sample from the five states. Approximately 22% of those surveyed have had their PM-JAY eligibility verified.

### Logistic regression results of determinants of verification

Table 2 shows the results the pooled multilevel logistic regression analyses with district level random effects.

**Table 2:** Pooled random effects logit regression results.

	Enrolment in PM-JAY	
Model	OR	95% CI (Bootstrap)
Individual Level Factors		
Relationship with Household Head		
*Household Head*	1.00	
*Spouse*	1.07	(1.00, 1.15)
*Other*	0.62^*^	(0.56, 0.68)
Sex		
*Male*	1.00	
*Female*	0.90^*^	(0.85, 0.95)
Age		
*0–15 years*	1.00	
*15–29 years*	1.43^*^	(1.31, 1.56)
*30–39 years*	1.98^*^	(1.77, 2.22)
*40–49 years*	2.18^*^	(1.89, 2.47)
*50–59 years*	2.61^*^	(2.22, 3.00)
*60 + years*	2.34^*^	(2.02, 2.76)
Unmarried		
*Married*	1.00	
*Unmarried*	1.14^*^	(1.04, 1.22)
Education Level Achieved		
*No formal education*	1.00	
*Up to Primary Level*	1.52^*^	(1.40, 1.64)
*Primary to Secondary Level*	1.95^*^	(1.78, 2.11)
*Above Secondary Level*	2.30^*^	(1.98, 2.68)
Illness requiring hospitalisation		
*Not hospitalised in the past year*	1.00	
*Hospitalised in the past year*	1.25^*^	(1.10, 1.42)
Has Chronic Condition		
*No Chronic Illness*	1.00	
*Has Chronic Illness*	1.17^*^	(1.07, 1.30)
Household Level Factors		
Household Size		
*1–5 members*	1.00	
*> 5 members*	0.88^*^	(0.81, 0.97)
Caste		
*General/Other or No Caste*	1.00	
*Scheduled Caste*	1.29^*^	(1.02, 1.64)
*Scheduled Tribe*	0.91	(0.72, 1.17)
*Other Backwards Classes*	1.48^*^	(1.20, 1.88)
Religion		
*Hindu*	1.00	
*Others*	1.33^*^	(1.13, 1.57)
Previous PFHI		
*Not enrolled in a PFHI*	1.00	
*Enrolled in a PFHI*	1.95^*^	(1.72, 2.23)
Household Consumption Quintile		
*1*^*st*^	1.00	
*2*^*nd*^	1.25^*^	(1.07, 1.45)
*3*^*rd*^	1.15	(0.97, 1.33)
*4*^*th*^	1.15	(0.96, 1.35)
*5*^*th*^ *(Highest quintile)*	1.27^*^	(1.06, 1.54)
Random Effects		
Districts		
Number	12	
Variance	1.57	
Residual Variance^†^	3.29	
ICC	0.32	
Hausman LL Test	0.00^*^	
Sample Size	50,002	

* p < 0.05.

†theoretical residual of π^2^/3 used for logistical regression.

The regression analysis suggests that variables at the individual and household level have a significant association with PM-JAY verification in line with the findings of the bivariate analysis (S1 Table). After adjusting for other sociodemographic variables and district random effects, several individual- and household-level characteristics were meaningfully associated with the likelihood of PM-JAY verification. We focus interpretation on the magnitude and direction of adjusted odds ratios (AORs) and their 95% confidence intervals (CIs), rather than on p-values alone.

At the individual level, when controlling for other sociodemographic variables and district random effects, there was little evidence of a meaningful difference in the likelihood of PM-JAY verification between household heads and their spouses (AOR 1.07, 95% CI 1.00–1.17), suggesting that heads and spouses have broadly similar verification probabilities after adjustment. Other members of households were less likely to have completed their verification than the household head, having approximately 38% lower odds of verification for non-head members relative to heads (AOR 0.62, 95% CI 0.56–0.68), indicating a substantively important disparity that may reflect differences in access to information, documents, or decision-making authority within households. Women had modestly lower odds of verification than men (AOR 0.90, 95% CI 0.85–0.95). While the relative difference is small, given the large number of eligible women, even this modest gap could translate into a sizeable absolute number of women not completing verification, with implications for equitable access to PM-JAY benefits. Unmarried individuals had slightly higher odds of verification than married individuals (AOR 1.14, 95% CI 1.04–1.22). The magnitude of this association is modest, but may point to differences in mobility, time availability, or responsibility for household administrative tasks between marital status groups. Older age was strongly associated with verification: individuals aged ≥60 years had more than twice the odds of verification compared with children aged 0–15 years (AOR 2.34, 95% CI 2.02–2.76). This pattern is consistent with higher perceived health needs among older adults and suggests that targeting younger age groups may be important to improve overall verification coverage. Educational gradients were pronounced: individuals with above-secondary education had more than double the odds of verification compared with those with no formal education (AOR 2.30, 95% CI 1.98–2.68). This indicates that lower education is a substantial barrier to verification and underscores the need for simplified procedures and targeted outreach for less-educated populations.

Individual illness history was also significantly associated with verification. Recent hospitalization in the past year was associated with moderately higher odds of verification (AOR 1.25, 95% CI 1.10–1.42), consistent with the expectation that contact with health services increases awareness of eligibility and motivation to complete verification. Similarly, individuals with chronic conditions had slightly higher odds of verification (AOR 1.18, 95% CI 1.07–1.30). Although these differences are modest, they suggest that people with greater health needs are somewhat more likely to engage with the verification process, but that many high-need individuals still remain unverified.

Household level factors were also important. Individuals in larger households (>5 members) had lower odds of verification than those in smaller households (AOR 0.88, 95% CI 0.81–0.97). This may reflect logistical challenges in coordinating documents and visits for multiple members, or dilution of attention and resources within larger households. Wealthier households were more likely to verify than the poorest: odds ratios increased from 1.15 in the fourth quintile to 1.27 in the fifth quintile relative to the poorest quintile (Q1). These gradients, while not large in relative terms, indicate that the poorest households (who are often the primary target of PM-JAY) face additional barriers to verification and may require enhanced support. Compared with general/no caste groups, scheduled castes (AOR 1.29, 95% CI 1.02–1.64) and other backward classes (AOR 1.48, 95% CI 1.20–1.88) had higher odds of verification, whereas scheduled tribes did not show a clear advantage. This pattern may reflect differential targeting, outreach, or administrative prioritization of certain socially disadvantaged groups, but also highlights that scheduled tribes are not benefiting proportionately and may need specific attention. Individuals belonging to religions other than Hinduism had moderately higher odds of verification (AOR 1.33, 95% CI 1.13–1.57). The reasons for this difference warrant further investigation, including possible differences in geographic distribution, community organization, or engagement with local implementation structures. Prior enrolment in a state PFHI scheme was strongly associated with verification (AOR 1.95, 95% CI 1.72–2.23), nearly doubling the odds. This suggests that familiarity with insurance processes and possibly pre-existing documentation substantially facilitate PM-JAY verification, and that first-time insurance populations may require additional navigation support.

District-level random effects accounted for approximately 32% of the variance in verification, indicating substantial heterogeneity across districts.

### Descriptive analysis of verification experience

Table 3 provides summary statistics of household head’s experience of the PM-JAY verification process. Common Service Centres were the most common location of verification in the sample (55%), compared to dedicated PM-JAY kiosks (15%) and empanelled hospitals (17%). The modal waiting time was greater than 45 minutes (40% of respondents), with respondents from Chhattisgarh (43%) and Gujarat (54%) reporting the longest waiting times. The number of documents required when verifying varied by state. Overall, most (56%) household heads required one or two documents. The majority of individuals had to pay a fee for a process that should be free according to PM-JAY guidelines. This fee ranged from 20 to 2,800 INR in this sample, with very high proportions observed in Gujarat (89%) and Meghalaya (90%). Nearly 38% of respondents who completed the verification process did not receive a Gold Card immediately after the completion of the process. A majority of respondents (83%) reported that they were satisfied with the verification process.

**Table 3:** Descriptive statistics of household head verification experiences.

Variable	Measure	Total (%)	Bihar (%)	Chhattisgarh (%)	Gujarat (%)	Meghalaya (%)	Uttar Pradesh (%)
Individual has experienced verification process	Yes	2,276 (23.1)	134 (5.2)	135 (9.1)	1,219 (71.3)	173 (11.5)	615 (23.8)
	No	7,596 (76.9)	2,455 (94.8)	1,353 (90.9)	490 (28.7)	1,330 (88.5)	1,968 (76.2)
Verification Location	Common Service Centre	1,248 (54.8)	67 (50.0)	28 (20.7)	869 (71.3)	22 (12.7)	262 (42.6)
	Empanelled Hospitals	390 (17.1)	13 (9.7)	72 (53.3)	56 (4.6)	93 (53.8)	156 (25.4)
	PM-JAY Kiosk	348 (15.3)	47 (35.1)	13 (9.6)	70 (5.7)	54 (31.2)	164 (26.7)
	Other	290 (12.7)	7 (5.2)	22 (16.3)	224 (18.4)	4 (2.3)	33 (5.4)
Waiting Times	<10 Minutes	199 (8.7)	22 (16.4)	18 (13.3)	61 (5.0)	25 (14.5)	73 (11.9)
	11-30 Minutes	533 (23.3)	51 (38.1)	29 (21.5)	218 (17.9)	36 (20.8)	199 (32.4)
	31-45 Minutes	478 (20.9)	8 (6.0)	24 (17.8)	268 (22.0)	44 (25.4)	134 (21.8)
	>45 Minutes	919 (40.2)	34 (25.4)	58 (43.0)	653 (53.6)	48 (27.7)	126 (20.5)
	Other	147 (6.4)	19 (14.2)	6 (4.4)	19 (1.6)	20 (11.6)	81 (13.2)
Number of Documents Required	1 Document	570 (25.0)	47 (35.1)	14 (10.4)	51 (4.2)	106 (61.3)	351 (57.1)
	2 Documents	707 (31.1)	73 (54.5)	54 (40.0)	311 (25.5)	42 (24.3)	227 (36.9)
	3 Documents	677 (29.7)	14 (10.4)	61 (45.2)	551 (45.2)	25 (14.5)	26 (4.2)
	4 Documents	316 (13.9)	0 (0.0)	6 (4.4)	301 (24.7)	0 (0.0)	9 (1.5)
	5 Documents	6 (~0.0)	0 (0.0)	0 (0.0)	4 (0.3)	0 (0.0)	2 (0.3)
Informal Payment Required	0 = No	676 (29.7)	44 (32.8)	114 (84.4)	138 (11.3)	17 (9.8)	363 (59.0)
	1 = Yes	1,600 (70.3)	90 (67.2)	21 (15.6)	1,081 (88.7)	156 (90.2)	252 (41.0)
Received Gold Card	0 = No	863 (37,9)	56 (41.8)	68 (50.4)	463 (38.0)	44 (25.4)	232 (37.7)
	1 = Yes	1,413 (62.1)	78 (58.2)	67 (49.6)	756 (62.0)	129 (74.6)	383 (62.3)
Satisfaction	0 = Unsatisfied	288 (12.7)	11 (8.2)	15 (11.1)	71 (5.8)	50 (28.9)	141 (22.9)
	1 = Satisfied	1,988 (87.3)	123 (91.8)	120 (88.9)	1148 (94.2)	123 (71.1)	474 (77.1)

## Discussion

Our study makes an important contribution to the literature by being the first large-scale, multi-state analysis of eligibility verification under PM-JAY. While PM-JAY was designed with an entitlement-based approach, in which cumbersome enrolment processes of earlier Indian PFHI were replaced by a simplified verification procedure and the elimination of beneficiary contributions to promote easier opting-in for beneficiaries, our results show that barriers persist, making the verification process ineffective in translating entitlement into uptake. First, more than a year after PM-JAY’s launch, only one-fifth of eligible individuals in our sample had completed verification. Second, verification was influenced by individual-level factors (household position, age, education, illness history, prior PFHI enrolment) and household-level factors (size, caste, religion, wealth). Notably, caste- and religion-based patterns suggested more equitable participation. Nearly one-third of the observed variation was attributable to district-level factors. Third, while most respondents expressed satisfaction with verification, detailed inquiry revealed procedural constraints and barriers. These findings suggest that despite PM-JAY’s entitlement design, beneficiaries continue to face inequities and operational challenges in verification, emphasizing the need for targeted reforms. Viewed through a universal health coverage lens, these verification gaps are important because they limit effective access to a publicly financed entitlement intended to reduce financial barriers for vulnerable households. The persistence of such gaps suggests that formal eligibility alone is insufficient to ensure equitable coverage.

Despite eliminating formal enrolment procedures and adopting entitlement-based eligibility, only about one-fifth of our sample had verified PM-JAY eligibility. These findings align with Mohanty et al. (2023), who reported PFHI coverage (measured as individual self-reported membership) of 38.6% in rural and 27.6% in urban areas during 2019–2021, although their estimates reflected combined national and state PFHI schemes rather than PM-JAY alone [21]. Our slightly lower estimates from 2020 likely result from focusing strictly on SECC-eligible households, whereas Mohanty et al. included additional beneficiaries under state eligibility adaptations and universal schemes in states such as Karnataka and Meghalaya. Further, the SECC data are from 2001, and many household’s socio-economic status may have changed since then, which is not reflected in PM-JAY targeting. Previous studies have shown higher enrolment under universal state PFHI, often driven by wealthier and more educated groups [22]. In neighbouring Pakistan, the province of Khyber Pakhtunkhwa has expanded the national Sehat Sahulat inpatient PFHI to universal coverage; however, entitlement has not translated into effective universal coverage, with similar challenges around beneficiary enrolment [23,24]. The observed shortfall in our findings may suggest gaps in reaching left-behind groups, which we further identify below, and for whom more targeted efforts are needed.

Historically, evidence indicates that enrolment in social health insurance and PFHI is strongly shaped by intra-household dynamics, power relations, and gender norms, influenced strongly by societal dynamics [25]. These disparities reflect broader, contextual gender inequities in healthcare access, costs, and financial protection, especially in the Indian context [26,27]. Our findings reflect similar patterns, showing that PM-JAY risks excluding women, household members who are not the head or spouse, and individuals with lower education and lower socio-economic status. We further discuss these findings, drawing comparisons primarily to studies on PFHI enrolment, given the paucity of studies on PFHI verification. We observed lower verification rates in household members other than the spouse or the head of the household and nearly 10% less verification in women compared to men. Both these patterns reflect differences in intra-household decision-making power [28] with household members occupying lesser decision-making positions under-represented (as evidenced by lower verification rates of younger individuals and among members from large families in our sample). Gender norms may restrict women’s awareness and information about PM-JAY [29] and limit their ability to travel to verification centers. Many social protection programs without explicit gender-sensitive provisions have been shown to perpetuate existing gender inequities [30]. Given this, PM-JAY’s gender-neutral design may be rendered “gender-blind”. For PM-JAY, design provisions such as gender-focused outreach, compulsory spousal verification (e.g., as for enrolment in RSBY) and compulsory verification of all household members could improve equity and overcome these blind spots. Further, verification challenges in large households may stem from requirements for physical presence, logistical barriers, or residual perceptions from earlier schemes. Ensuring universal verification within households may require outreach strategies or simplified procedures that eliminate the need for multiple members to be physically present.

We further found that higher education and wealth to be positively associated with verification, reinforcing international evidence on pro-rich enrolment patterns [31]. Given that our sample consisted of only PM-JAY eligible households, this suggests that inequities within this demographic still need to be addressed for egalitarian outreach. Contrary to earlier RSBY studies showing higher enrolment among Hindus and higher castes [32–34], we found that individuals from other religions were nearly 30% more likely to verify, and that socially disadvantaged groups (scheduled castes and other backward classes) had higher verification rates than general castes or those reporting no caste. These findings may indicate evolving patterns of social equity and sustained efforts are needed to tackle caste- and religion-based inequities, particularly when intersecting with gender, education, or wealth disadvantage [21,35,36]. For example, qualitative evidence from West Bengal highlights how marginalized caste women required connections with upper-caste actors to access RSBY enrolment [37], underlining the need for social protection programs to actively address this form of structural exclusion.

Individual illness history was also strongly associated with verification: those recently hospitalized or living with chronic conditions were more likely to verify, likely due to increased interaction with hospitals, which are key verification sites, and stronger incentives to secure coverage. Prior enrolment in state PFHI nearly doubled the odds of verification, suggesting positive spillovers. This contrasts with RSBY, where co-existing state PFHI was found to reduce enrolment [34]. The difference likely reflects structural changes under PM-JAY, which subsumes state schemes, eliminating competition and offering an opportunity to leverage prior scheme awareness to bolster verification uptake.

District-level factors explained nearly one-third of the variance in verification, highlighting the importance of geographical context in shaping PM-JAY uptake. Mohanty et al. similarly observed geographical inequalities in PFHI coverage, with increases observed among targeted rural households between 2015–2021, though not necessarily attributable to PM-JAY alone [21]. Differences across districts can be driven by variation in the timing, intensity, and content of awareness activities, as well as by structural and systemic capacities. State-level differences in PFHI enrolment have been highlighted elsewhere: Khan et al. (2021) found that state-level contextual factors explained more variation in coverage than community or district-level factors [38]. Comparable findings from Ethiopia attribute enrolment variation to regional disparities in “infrastructural power,” shaped by historical, political, and social factors, including gender norms [18,39,40]. For PM-JAY, district- and state-wise disparities have been linked to health system factors like uneven healthcare facility empanelment and density [41], variations in institutional capacity and leadership within facilities [42,43], and administrative capacity across states [42]. Closer in the region, infrastructural capacity differences across provinces and inadequate assistance for enrolment procedures have hindered enrolment in the Nepal Health Insurance Program [44–46] and in Pakistan’s Sehat Sahulat scheme [24]. In the Philippines, lack of health system infrastructure and capacity to implement verification procedures was reported to be a critical barrier for uptake [10]. These findings indicate that improving PM-JAY verification requires not only addressing individual- and household-level barriers but also strengthening state and district implementation capacity, infrastructure, and governance to ensure equitable reach. The Indian National Health Policy 2017 [47] outlines a roadmap for addressing specific gaps in health infrastructure and service capacity, while the Ayushman Bharat Digital Mission 2021 [48]promotes integrated and interoperable digital systems to improve service delivery; both initiatives can be aligned with PM-JAY processes to maximize equitable reach.

We also found substantial procedural variation in beneficiaries’ experiences with verification. Required documentation ranged from one to four documents; the necessity for official IDs and varied documentation requirements across states (which may not be known to beneficiaries) may reduce or deny access to groups like migrants who may lack them [49]. The Indian national AADHAAR biometric ID system [50], which is used across the country as a national identifier, can be one possible solution to replace the numerous documentation requirements; this may simplify identification and enable easier utilization of benefits across states. Further, most respondents reported waiting more than 45 minutes, indicating capacity constraints at verification centres, similar to experiences in the Philippines [10]. Strikingly, 70% reported making informal payments, highlighting the persistence of such entrenched practices within Indian healthcare [51]. While nearly 90% expressed satisfaction with the process, long waits, inconsistent documentation requirements, and informal charges suggest the need for reforms to make verification simpler and more responsive to beneficiaries. Patient-centred design approaches [52] and mechanisms for beneficiary feedback could strengthen engagement, while leveraging community-based or non-governmental actors (shown to be more trusted than government-led efforts in other LMIC contexts) [12] may enhance outreach.

While evidence from similar experiences with PFHI verification is scarce, international experience offers potential strategies to improve PFHI uptake, based primarily on efforts to increase enrolment. These include targeted information campaigns, use of beneficiary navigators, decentralised enrolment centres, and digital tools for awareness and reminders [6,49]. Evidence from India remains limited but promising: one randomized trial found that timing information campaigns closer to enrolment increased effectiveness by 60% [50], and other studies have shown positive effects of media exposure in promoting PFHI uptake, especially among marginalized castes [33,35]. Drawing from these findings and given PM-JAY’s high political visibility and extensive media coverage [12,51,52], more systematic use of mass and local media may be an especially powerful tool to boost verification and reach left-behind groups.

### Strengths and limitations

Our study draws on a large, district-representative sample of PM-JAY-eligible households across five diverse states, with information collected from all household members. The sampling frame was based on the 2011 SECC, consistent with PM-JAY’s eligibility criteria, ensuring representativeness of targeted households. However, the reliance on 2011 SECC data warrants further research to assess whether targeting effectively reaches current populations most in need of coverage. Our evidence is limited to rural areas, as urban households were not sampled. Data collection occurred within one year of PM-JAY’s launch, thus findings reflect the early implementation stages. Detailed enrolment experiences were obtained from only one household member, typically the head, hence intra-household variations, particularly among less powerful members, may not be fully captured. Further, while we document determinants of verification, we lack qualitative data on the motivations and barriers underlying insurance uptake decisions, which should be explored in future research. Finally, given the shortage of comparable evidence on verification processes in PFHI, we draw comparisons with the extensive literature on PFHI enrolment. While enrolment and verification are not synonymous, these comparisons are useful to understand how dynamics associated with enrolment compare with those for verification, as potentially more PFHI schemes transition to verification procedures.

## Conclusions

PM-JAY’s entitlement-based design sought to remove enrolment barriers, yet verification remains low and is influenced by individual, household, and district-level factors. We observe positive patterns among marginalized caste and other religion groups, and pro-rich and pro-education verification patterns. Reforms in scheme design which mandate gender-sensitive outreach by state agencies, ensure automatic or streamlined verification for all household members, and strengthen local health system capacity are needed in order to expand coverage. Future research should systematically investigate the motivations and barriers that shape decisions to take up health insurance, with particular attention to population groups currently left out of coverage, such as the poor, less educated populations and women. Building on these insights, studies should employ qualitative and participatory approaches to co-develop and test targeted strategies that effectively reach, engage, and retain these excluded groups in health insurance schemes. Government investments in district-level health infrastructure, administrative capacity, and community-based awareness campaigns can be useful in closing verification gaps. Addressing these structural and operational gaps is essential for PM-JAY to realize its mandate of effective universal health access and coverage for India’s poorest and most vulnerable.

## Supporting information

S1 Table Bivariate analysis and chi-squared results individual level analysis (N = 50,002). (DOCX)

## References

[1] Das J, Do QT. The prices in the crises: What we are learning from 20 years of health insurance in low- and middle-income countries. J Econ Perspect. 2023;37(2):123–52. doi: 10.1257/JEP.37.2.123

[2] Gottret P, Schieber G. Health financing revisited: A practitioner’s guide. In: Health financing revisited; 2006. doi: 10.1596/978-0-8213-6585-4

[3] Kwarteng A, Akazili J, Welaga P, Dalinjong PA, Asante KP, Sarpong D, et al. The state of enrollment on the National Health Insurance Scheme in rural Ghana after eight years of implementation. Int J Equity Health. 2019;19(1):4. doi: 10.1186/s12939-019-1113-0 31892331 PMC6938612

[4] Kusi A, Enemark U, Hansen KS, Asante FA. Refusal to enrol in Ghana’s National Health Insurance Scheme: Is affordability the problem? Int J Equity Health. 2015;14(1):1–14. doi: 10.1186/S12939-014-0130-2 25595036 PMC4300159

[5] Jehu-Appiah C, Aryeetey G, Agyepong I, Spaan E, Baltussen R. Household perceptions and their implications for enrollment in the National Health Insurance Scheme in Ghana. Health Policy Plan. 2012;27(3):222–33. doi: 10.1093/heapol/czr032 21504981

[6] James N, Acharya Y. Increasing health insurance enrollment in low- and middle-income countries: What works, what does not, and research gaps: A scoping review. Inquiry. 2022;59. doi: 10.1177/00469580221090396 35574923 PMC9121503

[7] Agustina R, Dartanto T, Sitompul R, Susiloretni KA, Suparmi, Achadi EL, et al. Universal health coverage in Indonesia: Concept, progress, and challenges. Lancet. 2019;393(10166):75–102. doi: 10.1016/S0140-6736(18)31647-7 30579611

[8] Mahardika I, Marliani L, Pratama P, Salame HS, Samudra R, Wakhidah N, et al. The challenges of universal health insurance in developing countries: Experimental evidence from Indonesia’s national health insurance. Am Econ Rev. 2021;111(9):3035–63. doi: 10.1257/AER.20200523

[9] Akweongo P, Gadeka DD, Aryeetey G, Sumboh J, Aheto JMK, Aikins M, et al. Does mobile renewal make health insurance more responsive to clients? A case study of the National Health Insurance Scheme in Ghana. BMJ Glob Health. 2023;7(Suppl 6):e011440. doi: 10.1136/bmjgh-2022-011440 38148107 PMC10846841

[10] Co PA, Garcia GL, Samson MC, Alvior RK, Mariano MA, Boxshall M. Universal health coverage on paper and in practice: Implementing the immediate eligibility policy of the Philippine health insurance system (PhilHealth). 2025.

[11] NHA. About Pradhan Mantri Jan Arogya Yojana. National Health Authority; 2019.

[12] Srivastava S, Bertone MP, Parmar D, Walsh C, De Allegri M. The genesis of the PM-JAY health insurance scheme in India: Technical and political elements influencing a national reform towards universal health coverage. Health Policy Plan. 2023;38(7):862–75. doi: 10.1093/heapol/czad045 37436821

[13] Virk AK, Atun R. Towards universal health coverage in India: A historical examination of the genesis of Rashtriya Swasthya Bima Yojana - The health insurance scheme for low-income groups. Public Health. 2015;129(6):810–7. doi: 10.1016/j.puhe.2015.02.002 25753280

[14] Kumar A, Sarwal R. Health insurance for India’s missing middle. 2021.

[15] About the 2030 Agenda on Sustainable Development. OHCHR [Internet]. [cited 2026 Jun 9]. Available from: https://www.ohchr.org/en/sdgs/about-2030-agenda-sustainable-development

[16] Government of India. Beneficiary Identification Guidelines Ayushman Bharat-Pradhan Mantri Jan Arogya Yojana (AB PM-JAY). 2023.

[17] De Allegri M, Srivastava S, Strupat C, Brenner S, Parmar D, Parisi D, et al. Mixed and multi-methods protocol to evaluate implementation processes and early effects of the Pradhan Mantri Jan Arogya Yojana scheme in seven Indian states. Int J Environ Res Public Health. 2020;17(21):7812. doi: 10.3390/ijerph17217812 33114480 PMC7663328

[18] Lavers T. Aiming for Universal Health Coverage through insurance in Ethiopia: State infrastructural power and the challenge of enrolment. Soc Sci Med. 2021;282:114174. doi: 10.1016/j.socscimed.2021.114174 34175571

[19] NSSO. Key indicators of social consumption in India: Health. National Sample Survey Organisation [Internet]; 2015. Available from: http://mail.mospi.gov.in/index.php/catalog/161/download/1949

[20] Government of India. Beneficiary Identification Guidelines Ayushman Bharat-Pradhan Mantri Jan Arogya Yojana (AB PM-JAY).

[21] Mohanty SK, Upadhyay AK, Maiti S, Mishra RS, Kämpfen F, Maurer J, et al. Public health insurance coverage in India before and after PM-JAY: Repeated cross-sectional analysis of nationally representative survey data. BMJ Glob Health. 2023;8(8):e012725. doi: 10.1136/BMJGH-2023-012725 37640493 PMC10462969

[22] Dutta EK, Kumar S, Venkatachalam S, Downey LE, Albert S. An analysis of government-sponsored health insurance enrolment and claims data from Meghalaya: Insights into the provision of health care in North East India. PLoS One. 2022;17(6):e0268858. doi: 10.1371/journal.pone.0268858 35657934 PMC9165869

[23] Sen G, Govender V, El-Gamal S. Universal health coverage, gender equality and social protection: A health systems approach. 38. United Nations Entity for Gender Equality and the Empowerment of Women, UN Women; 2020.

[24] Moradhvaj, Saikia N. Gender disparities in health care expenditures and financing strategies (HCFS) for inpatient care in India. SSM Popul Health. 2019;9:100372. doi: 10.1016/j.ssmph.2019.100372 31998823 PMC6978493

[25] Kumar K, Singh A, James KS, McDougal L, Raj A. Gender bias in hospitalization financing from borrowings, selling of assets, contribution from relatives or friends in India. Soc Sci Med. 2020;260:113222. doi: 10.1016/j.socscimed.2020.113222 32707443 PMC7441313

[26] Zegeye B, Idriss-Wheeler D, Ahinkorah BO, Ameyaw EK, Seidu A-A, Adjei NK, et al. Association between women’s household decision-making autonomy and health insurance enrollment in sub-Saharan Africa. BMC Public Health. 2023;23(1):610. doi: 10.1186/s12889-023-15434-z 36997885 PMC10064715

[27] RamPrakash R, Lingam L. Why is women’s utilization of a publicly funded health insurance low?: A qualitative study in Tamil Nadu, India. BMC Public Health. 2021;21(1):350. doi: 10.1186/s12889-021-10352-4 33579249 PMC7881649

[28] Perera C, Bakrania S, Ipince A, Nesbitt-Ahmed Z, Obasola O, Richardson D, et al. Impact of social protection on gender equality in low- and middle-income countries: A systematic review of reviews. Campbell Syst Rev. 2022:e1240. doi: 10.1002/cl2.1240 PMC9133545 36913187

[29] Osei Afriyie D, Krasniq B, Hooley B, Tediosi F, Fink G. Equity in health insurance schemes enrollment in low and middle-income countries: A systematic review and meta-analysis. Int J Equity Health. 2022;21(1):21. doi: 10.1186/s12939-021-01608-x 35151323 PMC8841076

[30] Aashima, Sharma R. Inequality and disparities in health insurance enrolment in India. J Med Surg Public Health. 2023;1:100009. doi: 10.1016/j.glmedi.2023.100009

[31] Yadav MK, Mohanty PC. Determinants of choice of health insurance: Empirical evidence from responses of Indian households. Int J Health Plan Manag. 2021;36(5):1809–29. doi: 10.1002/HPM.3267 34169579

[32] Nandi A, Ashok A, Laxminarayan R. The socioeconomic and institutional determinants of participation in India’s health insurance scheme for the poor. PLoS One. 2013;8(6):e66296. doi: 10.1371/journal.pone.0066296 23805211 PMC3689788

[33] Ambade PN, Pakhale S, Rahman T. Explaining caste-based disparities in enrollment for national health insurance program in India: A decomposition analysis. J Racial Ethn Health Disparities. 2023;10(4):1918–32. doi: 10.1007/s40615-022-01374-8 35994172

[34] Sharma SK, Vishwakarma D, Puri P. Gender disparities in the burden of non-communicable diseases in India: Evidence from the cross-sectional study. Clin Epidemiol Glob Health. 2020;8(2):544–9. doi: 10.1016/j.cegh.2019.11.011

[35] Sen K, Gupta S. Masking poverty and entitlement: RSBY in selected districts of West Bengal. Soc Change. 2017;47(3):339–58. doi: 10.1177/0049085717715557

[36] Khan PK, Perkins JM, Kim R, Mohanty SK, Subramanian SV. Multilevel population and socioeconomic variation in health insurance coverage in India. Trop Med Int Health. 2021;26(10):1285–95. doi: 10.1111/tmi.13645 34181806

[37] Mann M. The autonomous power of the state: Its origins, mechanisms and results. Eur J Sociol. 1984;25(2):185–213. doi: 10.1017/s0003975600004239

[38] Soifer H. State infrastructural power: Approaches to conceptualization and measurement. Stud Comp Int Dev. 2008;43(3–4):231–51. doi: 10.1007/S12116-008-9028-6

[39] Joseph J, Sankar D H, Nambiar D. Empanelment of health care facilities under Ayushman Bharat Pradhan Mantri Jan Arogya Yojana (AB PM-JAY) in India. PLoS One. 2021;16(5):e0251814. doi: 10.1371/journal.pone.0251814 34043664 PMC8158976

[40] Srivastava S, Bertone MP, Basu S, De Allegri M, Brenner S. Implementation of PM-JAY in India: A qualitative study exploring the role of competency, organizational and leadership drivers shaping early roll-out of publicly funded health insurance in three Indian states. Health Res Policy Syst. 2023;21(1):65. doi: 10.1186/s12961-023-01012-7 37370159 PMC10294452

[41] Saxena A, Trivedi M, Shroff ZC, Sharma M. Improving hospital-based processes for effective implementation of Government funded health insurance schemes: Evidence from early implementation of PM-JAY in India. BMC Health Serv Res. 2022;22(1):73. doi: 10.1186/s12913-021-07448-3 35031024 PMC8760668

[42] Government of India. National Health Policy, 2017. 31 [Internet]; 2017. Available from: http://cdsco.nic.in/writereaddata/National-Health-Policy.pdf

[43] NHA | Official website Ayushman Bharat Digital Mission [Internet]. [cited 2023 Aug 14]. Available from: https://abdm.gov.in/

[44] Exclusion by design: How national ID systems make social protection inaccessible to vulnerable populations. Privacy International [Internet]. [cited 2025 Dec 5]. Available from: https://privacyinternational.org/long-read/4472/exclusion-design-how-national-id-systems-make-social-protection-inaccessible

[45] Abraham R, Bennett ES, Sen N, Shah NB. State of Aadhaar Report 2016–17 [Internet]; 2016 [cited 2026 Apr 2]. Available from: http://stateofaadhaar.in

[46] Selvaraj S, Karan AK, Srivastava S, Bhan N, Mukhopadhyay I. India health system review, vol. 11. New Delhi: World Health Organization, Regional Office for South-East Asia; 2022.

[47] Meskó B, de Bronkart D. Patient design: The importance of including patients in designing health care. J Med Internet Res. 2022;24(8):e39178. doi: 10.2196/39178 36044250 PMC9475404

[48] Ismail A, Mathauer I, Akweongo P, Bolivar Vargas MC, Desai S, Raharja DP, et al. Making health insurance responsive to citizens: Learning from six low-income and middle-income countries. BMJ Glob Health. 2025;7(Suppl 6):e018176. doi: 10.1136/bmjgh-2024-018176 40409763 PMC12107628

[49] Capuno JJ, Kraft AD, Quimbo S, Tan CR, Wagstaff A. Effects of interventions to raise voluntary enrollment in a social health insurance scheme: A cluster randomized trial. The World Bank; 2014. (Policy Research Working Papers). doi: 10.1596/1813-9450-6893

[50] Das J, Leino J. Evaluating the RSBY: Lessons from an experimental information campaign. Econ Polit Wkly. 2011;xlvi(32):85–93.

[51] Kalita A, Croke K. The politics of health policy agenda setting in India: The case of the PMJAY program. Health Syst Reform. 2023;9(1):2229062. doi: 10.1080/23288604.2023.2229062 37432137

[52] Lahariya C. “Ayushman Bharat” program and universal health coverage in India. Indian Pediatr. 2018;55(6):495–506. doi: 10.1007/s13312-018-1341-1 29978817

