# Supplementary material for: Bridging the gap between health insurance eligibility and uptake: A multistate quantitative analysis of entitlement-based verification patterns under India’s PM-JAY Scheme
Source: PLOS Glob Public Health. 2026 Oct 13;6(10):e0007451. doi: 10.1371/journal.pgph.0007451 (PMC13660894; doi:10.1371/journal.pgph.0007451)
Supplement: Supplementary file 1 [file pgph.0007451.s001.docx]

***Bivariate Analysis***

S1 Table: Bivariate Analysis and Chi-Squared Results Individual Level Analysis (N=50,002)

| Variable | PM-JAY Enrolment  Verified | | Total | |  | | |
| --- | --- | --- | --- | --- | --- | --- | --- |
|  | % | N | % | N | | Chi-square value | P-value |
| Individual Level Variables |  |  |  |  | |  |  |
| *Relationship to Head of Household* |  |  |  |  | | 879.186 | 0.000 |
| Household Head | 27.6 | 3,034 | 19.6 | 9,804 | |  |  |
| Spouse | 18.7 | 2,050 | 15.0 | 7,519 | |  |  |
| Other | 53.7 | 5,894 | 65.4 | 32,679 | |  |  |
| Total | 100.0 | 10,978 | 100.0 | 50,002 | |  |  |
| *Sex* |  |  |  |  | | 31.430 | 0.000 |
| Male | 52.7 | 5,786 | 50.3 | 25,172 | |  |  |
| Female | 47.3 | 5,192 | 49.7 | 24,830 | |  |  |
| Total | 100.0 | 10,978 | 100.0 | 50,002 | |  |  |
| Age |  |  |  |  | | 842.857 | 0.000 |
| 0-15 years | 19.9 | 2,189 | 33.3 | 16,637 | |  |  |
| 16-29 years | 25.7 | 2,821 | 25.6 | 12,785 | |  |  |
| 30-39 years | 14.9 | 1,635 | 12.1 | 6,051 | |  |  |
| 40-49 years | 15.5 | 1,703 | 11.2 | 5,621 | |  |  |
| 50-59 years | 11.3 | 1,245 | 7.7 | 3,839 | |  |  |
| 60+ years | 12.6 | 1,385 | 10.1 | 5,039 | |  |  |
| Total | 100.0 | 10,978 | 100.0 | 50,002 | |  |  |
| *Marital Status* |  |  |  |  | | 545.617 | 0.000 |
| Married | 54.0 | 5,931 | 44.2 | 22,123 | |  |  |
| Not Married | 46.0 | 5,047 | 55.8 | 27,879 | |  |  |
| Total | 100.0 | 10,978 | 100.0 | 50,002 | |  |  |
| *Education* |  |  |  |  | | 282.241 | 0.000 |
| Illiterate | 33.0 | 3,624 | 37.7 | 18,859 | |  |  |
| Up to Primary | 23.3 | 2,558 | 25.3 | 12,666 | |  |  |
| Up to Secondary | 37.8 | 4,154 | 32.3 | 16,150 | |  |  |
| Above Secondary | 5.8 | 642 | 4.7 | 2,327 | |  |  |
| Total | 100.0 | 10,978 | 100.0 | 50,002 | |  |  |
| *Hospitalisation* |  |  |  |  | | 44.541 | 0.000 |
| Not hospitalised in past year | 95.4 | 10,478 | 96.5 | 48,243 | |  |  |
| Hospitalised in the past year | 4.6 | 500 | 3.5 | 1,759 | |  |  |
| Total | 100.0 | 10,978 | 100.0 | 50,002 | |  |  |
| *Chronic Illness* |  |  |  |  | | 65.636 | 0.000 |
| No Chronic Illness | 90.7 | 9,954 | 92.5 | 46,239 | |  |  |
| Has Chronic Illness | 9.3 | 1,024 | 7.5 | 3,763 | |  |  |
| Total | 100.0 | 10,978 | 100.0 | 50,002 | |  |  |
| Household Level Variables |  |  |  |  | | 105.244 | 0.000 |
| *Household Size* |  |  |  |  | |  |  |
| 1-5 members | 47.8 | 5,244 | 43.5 | 21,741 | |  |  |
| 6+ members | 52.2 | 5,734 | 56.5 | 28,261 | |  |  |
| Total | 100.0 | 10,978 | 100.0 | 50,002 | |  |  |
| *Caste* |  |  |  |  | | 535.973 | 0.000 |
| Scheduled Caste | 23.2 | 2,545 | 32.0 | 16,016 | |  |  |
| Scheduled Tribe | 34.9 | 3,828 | 30.1 | 15,066 | |  |  |
| Other Backward Class | 36.3 | 3,988 | 31.9 | 15,964 | |  |  |
| General/Other or No Caste | 5.6 | 617 | 5.9 | 2,956 | |  |  |
| Total | 100.0 | 10,978 | 100.0 | 50,002 | |  |  |
| *Religion* |  |  |  |  | | 132.335 | 0.000 |
| Hindu | 75.9 | 8,337 | 79.8 | 39,921 | |  |  |
| Others | 24.1 | 2,641 | 20.2 | 10,081 | |  |  |
| Total | 100.0 | 10,978 | 100.0 | 50,002 | |  |  |
| *Other Publicly Funded Health Insurance (PFHI)* |  |  |  |  | | 10.085 | 0.002 |
| No prior enrolment in PFHI | 51.6 | 5,661 | 50.2 | 25,115 | |  |  |
| Prior enrolment in PFHI | 48.4 | 5,317 | 49.8 | 24,887 | |  |  |
| Total | 100.0 | 10,978 | 100.0 | 50,002 | |  |  |
| *Yearly Household Consumption Expenditure Quintiles* |  |  |  |  | | 497.445 | 0.000 |
| 1^st^ Quintile | 13.7 | 1,504 | 20.0 | 10,001 | |  |  |
| 2^nd^ Quintile | 18.7 | 2,058 | 20.0 | 9,990 | |  |  |
| 3^rd^ Quintile | 20.2 | 2,214 | 20.0 | 10,005 | |  |  |
| 4^th^ Quintile | 22.7 | 2,493 | 20.0 | 9,996 | |  |  |
| 5^th^ Quintile | 24.7 | 2,709 | 20.0 | 10,000 | |  |  |
| Total | 100.0 | 10,978 | 100.0 | 50,002 | |  |  |
| Household Location |  |  |  |  | |  |  |
| *District* |  |  |  |  | | 7,820.763 | 0.000 |
| District A, Bihar | 1.7 | 183 | 10.7 | 5,335 | |  |  |
| District B, Bihar | 1.2 | 127 | 7.5 | 3,750 | |  |  |
| District C, Bihar | 6.2 | 686 | 9.6 | 4,788 | |  |  |
| District D, Chhattisgarh | 2.7 | 294 | 7.0 | 3,476 | |  |  |
| District E, Chhattisgarh | 1.4 | 156 | 5.7 | 2,849 | |  |  |
| District F, Gujarat | 19.9 | 2,186 | 8.0 | 4,013 | |  |  |
| District G, Gujarat | 17.4 | 1,912 | 8.0 | 4,016 | |  |  |
| District H, Meghalaya | 7.0 | 767 | 7.1 | 3,555 | |  |  |
| District I, Meghalaya | 11.8 | 1,297 | 8.4 | 4,219 | |  |  |
| District J, Uttar Pradesh | 11.5 | 1,257 | 8.3 | 4,169 | |  |  |
| District K, Uttar Pradesh | 4.5 | 495 | 9.4 | 4,717 | |  |  |
| District L, Uttar Pradesh | 14.7 | 1,618 | 10.2 | 5,115 | |  |  |
| Total | 100.0 | 10,978 | 100.0 | 50,002 | |  |  |
