# Supplementary material for: Bridging the gap between health insurance eligibility and uptake: A multistate quantitative analysis of entitlement-based verification patterns under India’s PM-JAY Scheme
Source: PLOS Glob Public Health. 2026 Oct 13;6(10):e0007451. doi: 10.1371/journal.pgph.0007451 (PMC13660894; doi:10.1371/journal.pgph.0007451)
Supplement: Supplementary file 6 [file pgph.0007451.s007.docx]

September 21, 2026

Dear Dr Pacheco and Reviewers,

We thank you for the thoughtful reviews of our manuscript and your encouraging remarks on the revisions made. We sincerely thank Reviewers #1 and #2 for their encouraging feedback and are pleased to have addressed all their comments.

We have carefully considered the additional comments from Reviewer #3 and have tried to best address them. We feel that the manuscript has greatly improved as a result of this. Below we provide a point-by-point response, and the corresponding edits which have been made in the manuscript.

We welcome any further suggestions you may have.

Kind regards,

All authors

**Response to Reviewer** #**3**

*Reviewer #3: As the statistical reviewer I will focus on methods and reporting. Note that this is the first time I see this paper.*

*Major*

*1) The manuscript should describe how missing data were handled in the analysis. At present, it is not clear whether a complete-case analysis was used, whether any observations were excluded because of missing covariates, or what the level of missingness was for each variable. The authors should report missingness clearly, ideally by variable, and consider a sensitivity analysis using multiple imputation if missingness is non-trivial.*

Thank you for this helpful comment. We now describe how we handled missing data and reported the exact number of dropped households and final participants in the revised manuscript. As the number of missing households was small (0.4% of all households in the original dataset), we did not perform any imputation for missing values. We have included the following additions in Lines 217-225:

“21 households corresponding to 108 individuals from the original dataset were dropped from the analysis due to incomplete household consumption data or missing age of individual household members. 3 individuals were excluded as they identified as transgender, so we did not have a sufficient sample to comment on this gender group. 55 individuals were removed for having an unknown age. A further 147 were excluded as they responded that they were enrolled in a government health insurance scheme, which should have disqualified them for PM-JAY. As the number of missing households was small (0.4% of all households in the original dataset), and the number of excluded individuals in total was small (313, 0.6% of all individuals) we did not perform any imputation for missing values. The final dataset included 9,833 households (50,002 individuals). From these households, the 2,276 household heads (or another available adult member) who knew about PM-JAY, were eligible for the program, and had undergone the verification process were asked detailed questions about their experiences.”

*2) The modelling approach is broadly appropriate for a binary outcome, and the use of district-level random effects is reasonable given likely differences in implementation across districts. However, the analysis is conducted at the individual level while individuals are nested within households, so the authors should clarify whether household-level clustering was accounted for, and if not, consider this or justify its omission. The model should also more clearly state which covariates were included and why. The bivariate analyses appear mainly descriptive, the authors should clarify their purpose and confirm that univariable p-values were not used for model selection*

We thank the reviewer for this thoughtful comment on clustering and model specification. In response, we have: (i) clarified that our primary model accounts for district-level clustering via random effects but does not include a household-level random effect, and we justify this choice on substantive and statistical grounds; (ii) made adjustments in our estimations of standard errors by accounting for household level clustering using bootstrap estimations of the distribution (this has been added to the methods section, and the confidence intervals estimated have been updated in the results table and throughout the manuscript) (iii) made the covariate list and rationale more explicit; and (iv) clarified the purpose of the bivariate analyses and confirmed that univariable p-values were not used for model selection. The relevant Methods text has been revised accordingly:

Lines 267-270: “These bivariate analyses were descriptive and intended to summarize the distribution of key covariates by verification status and to identify patterns for presentation in Table 1; they were not used for variable selection in the multivariable model.”

Lines 279-295: “As individuals are nested within households, which in turn are nested within districts, we adjusted the standard errors of the regression model estimates to account for household level clustering using a nonparametric cluster bootstrap run 500 times. We resampled with replacement from the observed data at the household level, refit the model on each resampled dataset to obtain a distribution of coefficient estimates, and constructed 95% confidence intervals for each fixed-effect coefficient using the percentile method.

We did not add a household-level random effect because our substantive focus is on individual- and household-level predictors rather than household-specific latent effects, we therefore treat the district random effect as the primary clustering adjustment and acknowledge the possibility of residual within-household correlation as a limitation.

All covariates included in the multivariable model were specified a priori based on conceptual frameworks of health insurance uptake and our hypotheses described above. The model included the following covariates: household size; MPCE quintile; caste; religion; relationship to household head; sex; age group; marital status; education; chronic illness in the last 3 months; hospitalization in the past year; and prior coverage by a state PFHI. These variables were retained regardless of their statistical significance in bivariate or univariable analyses to avoid selection bias and to ensure adjustment for key confounders identified in the literature.”

*3) The results place considerable emphasis on statistical significance, including repeated use of p-values. As the sample size is large, small differences may become statistically significant without necessarily being substantively important. The authors should place greater emphasis on the size and direction of associations, and the uncertainty around estimates, and interpret the findings in terms of practical or policy relevance rather than statistical significance alone.*

We thank the reviewer for this important comment. We agree that, given the large sample size, overemphasis on statistical significance can be misleading. In the revised manuscript, we have: (i) reduced repetitive reporting of p-values and removed language that equates “significant” with “important”; (ii) highlighted the magnitude, direction, and confidence intervals of associations; and (iii) added interpretation of key findings in terms of practical and policy relevance for PM-JAY implementation. The relevant Results text has been fully revised accordingly:

Lines 319-401: “After adjusting for other sociodemographic variables and district random effects, several individual- and household-level characteristics were meaningfully associated with the likelihood of PM-JAY verification. We focus interpretation on the magnitude and direction of adjusted odds ratios (AORs) and their 95% confidence intervals (CIs), rather than on p-values alone.

At the individual level, when controlling for other sociodemographic variables and district random effects, there was little evidence of a meaningful difference in the likelihood of PM-JAY verification between household heads and their spouses (AOR 1.07, 95% CI 1.00–1.17), suggesting that heads and spouses have broadly similar verification probabilities after adjustment. Other members of households were less likely to have completed their verification than the household head, having approximately 38% lower odds of verification for non-head members relative to heads (AOR 0.62, 95% CI 0.56–0.68), indicating a substantively important disparity that may reflect differences in access to information, documents, or decision-making authority within households. Women had modestly lower odds of verification than men (AOR 0.90, 95% CI 0.85–0.95). While the relative difference is small, given the large number of eligible women, even this modest gap could translate into a sizeable absolute number of women not completing verification, with implications for equitable access to PM-JAY benefits. Unmarried individuals had slightly higher odds of verification than married individuals (AOR 1.14, 95% CI 1.04–1.22). The magnitude of this association is modest, but may point to differences in mobility, time availability, or responsibility for household administrative tasks between marital status groups. Older age was strongly associated with verification: individuals aged ≥60 years had more than twice the odds of verification compared with children aged 0–15 years (AOR 2.34, 95% CI 2.02–2.76). This pattern is consistent with higher perceived health needs among older adults and suggests that targeting younger age groups may be important to improve overall verification coverage. Educational gradients were pronounced: individuals with above-secondary education had more than double the odds of verification compared with those with no formal education (AOR 2.30, 95% CI 1.98–2.68). This indicates that lower education is a substantial barrier to verification and underscores the need for simplified procedures and targeted outreach for less-educated populations.

Individual illness history was also significantly associated with verification. Recent hospitalization in the past year was associated with moderately higher odds of verification (AOR 1.25, 95% CI 1.10–1.42), consistent with the expectation that contact with health services increases awareness of eligibility and motivation to complete verification. Similarly, individuals with chronic conditions had slightly higher odds of verification (AOR 1.18, 95% CI 1.07–1.30). Although these differences are modest, they suggest that people with greater health needs are somewhat more likely to engage with the verification process, but that many high-need individuals still remain unverified.

Household level factors were also important. Individuals in larger households (>5 members) had lower odds of verification than those in smaller households (AOR 0.88, 95% CI 0.81–0.97). This may reflect logistical challenges in coordinating documents and visits for multiple members, or dilution of attention and resources within larger households. Wealthier households were more likely to verify than the poorest: odds ratios increased from 1.15 in the fourth quintile to 1.27 in the fifth quintile relative to the poorest quintile (Q1). These gradients, while not large in relative terms, indicate that the poorest households (who are often the primary target of PM-JAY) face additional barriers to verification and may require enhanced support. Compared with general/no caste groups, scheduled castes (AOR 1.29, 95% CI 1.02–1.64) and other backward classes (AOR 1.48, 95% CI 1.20–1.88) had higher odds of verification, whereas scheduled tribes did not show a clear advantage. This pattern may reflect differential targeting, outreach, or administrative prioritization of certain socially disadvantaged groups, but also highlights that scheduled tribes are not benefiting proportionately and may need specific attention. Individuals belonging to religions other than Hinduism had moderately higher odds of verification (AOR 1.33, 95% CI 1.13–1.57). The reasons for this difference warrant further investigation, including possible differences in geographic distribution, community organization, or engagement with local implementation structures. Prior enrolment in a state PFHI scheme was strongly associated with verification (AOR 1.95, 95% CI 1.72–2.23), nearly doubling the odds. This suggests that familiarity with insurance processes and possibly pre-existing documentation substantially facilitate PM-JAY verification, and that first-time insurance populations may require additional navigation support.

District-level random effects accounted for approximately 32% of the variance in verification, indicating substantial heterogeneity across districts.”

*Minor*

*1) The manuscript does not state whether reporting followed an appropriate checklist, such as STROBE for observational studies, and I could not see a completed checklist among the submitted materials.*

Thank you for this comment. We have included a completed STROBE Statement—Checklist of items that should be included in reports of cross-sectional studies and have included the following statement in the methods section:

Lines 301-302: “Reporting of all items in this study adhered to the STROBE statement for reports of cross-sectional studies.”
