# Supplementary material for: Bridging the gap between health insurance eligibility and uptake: A multistate quantitative analysis of entitlement-based verification patterns under India’s PM-JAY Scheme
Source: PLOS Glob Public Health. 2026 Oct 13;6(10):e0007451. doi: 10.1371/journal.pgph.0007451 (PMC13660894; doi:10.1371/journal.pgph.0007451)
Supplement: Supplementary file 7 [file pgph.0007451.s002.docx]

**Breaking down barriers to enrolment in health insurance: a multistate quantitative analysis of entitlement-based verification patterns under India’s PM-JAY Scheme**

**Background**

The authors gave an insight to the issues of health insurance enrolment in the PM-JAY scheme in India. The discussion is well presented as it gave some examples from the LMICs. However, on Line 110, plural form of evidence should be used to “evidences show” or “evidence show”.

**Methods**

The method adopted for the study is apt and discussed in detail. Meanwhile, I do not have the expertise to comment on the model adopted. It is my hope that other reviewers will provide relevant feedback in that regard. However, most of the analyses are clear.

**Discussion**

The discussion section is well presented citing related previous studies.

**Conclusion**

The conclusion section is concise but it is necessary to give more actionable recommendation talking about who does what.

**Decision:** Accept with minor revision subject to Editor’s satisfaction.

**Date:** 14/04/2026

Regards,

**Afeez Lawal, Ph.D.**
