# Supplementary material for: Bridging the gap between health insurance eligibility and uptake: A multistate quantitative analysis of entitlement-based verification patterns under India’s PM-JAY Scheme
Source: PLOS Glob Public Health. 2026 Oct 13;6(10):e0007451. doi: 10.1371/journal.pgph.0007451 (PMC13660894; doi:10.1371/journal.pgph.0007451)
Supplement: Supplementary file 8 [file pgph.0007451.s003.docx]

The manuscript titled *“Breaking down barriers to enrolment in health insurance: a multistate quantitative analysis of entitlement-based verification patterns under India’s PM-JAY Scheme”* addresses a highly relevant issue in both national and global health policy contexts. The focus on entitlement-based verification under PM‑JAY is timely and potentially impactful not only for India but other countries also. However, I have some observations and suggestions that may add value to the manuscript:

1. Clarity of Objective in the Abstract
   The authors aim to investigate the determinants of undergoing verification among PM‑JAY’s target population in order to identify underserved groups and inform strategies to expand coverage. This central objective should be explicitly and clearly stated in the abstract to help readers quickly grasp the study’s purpose.
2. Presentation of Key Quantitative Findings in the Abstract
   As this is a quantitative study, the abstract should report key statistical findings. This may include enrolment or verification status proportions, major associated factors, and statistically significant predictors of enrolment/verification. Presenting these results will enhance the informativeness and completeness of the abstract.
3. Historical Context in the Introduction
   The introduction would benefit from a brief overview of the historical development of health insurance in India. This will provide important context for the emergence of PM‑JAY and situate the study within the broader evolution of India’s health financing landscape.
4. Strengthening the Methods Section
   The Methods section requires additional detail and standardization:

- A brief description of the research instrument/tool used, including its development and validation process, should be added.
- Please align the reporting more closely with STROBE guidelines for observational studies, including:
  - Sample size calculation and rationale
  - Sampling method and procedures
  - Inclusion and exclusion criteria
  - Tools Development and its validation processes
  - Respondent selection process
  - Quality assurance measures
- Clearly explain the rationale for the chosen age-group categorization.
- Provide a precise description of how household consumption (HCQ) was measured and operationalized.

5. Philosophical and Theoretical Linkage
   Incorporating a philosophical or theoretical framework (such as social justice, health equity theories) would strengthen the analytical depth of the study and better justify the interpretation of findings.
6. Policy Context
   The manuscript should explicitly reference relevant national policies related to PM‑JAY, health insurance, and Universal Health Coverage (UHC). This will enhance policy relevance and clarify how the findings contribute to ongoing health system reforms in India.
7. Alignment Between Title and Content
   Upon reviewing the full text, there appears to be a partial mismatch between the article title and the content presented. The authors are encouraged to reconsider whether the title accurately reflects the scope, focus, and analyses described in the manuscript, and to revise it if necessary for better alignment.
