# Supplementary material for: Bridging the gap between health insurance eligibility and uptake: A multistate quantitative analysis of entitlement-based verification patterns under India’s PM-JAY Scheme
Source: PLOS Glob Public Health. 2026 Oct 13;6(10):e0007451. doi: 10.1371/journal.pgph.0007451 (PMC13660894; doi:10.1371/journal.pgph.0007451)
Supplement: Supplementary file 9 [file pgph.0007451.s005.docx]

June 9, 2026

Dear Reviewers and Editors,

We thank you for the thoughtful reviews of our manuscript and your encouraging remarks on the revisions made. We have carefully considered the additional, detailed comments and have tried to best incorporate them into the manuscript. We feel that the manuscript has greatly improved as a result of this.

Below we provide a point-by-point response, and the corresponding edits which have been made in the manuscript.

We welcome any further suggestions you may have.

Kind regards,

All authors

**Response to Reviewer 1**

**Breaking down barriers to enrolment in health insurance: a multistate quantitative analysis of entitlement-based verification patterns under India’s PM-JAY Scheme**

**Background**

The authors gave an insight to the issues of health insurance enrolment in the PM-JAY scheme in India. The discussion is well presented as it gave some examples from the LMICs. However, on Line 110, plural form of evidence should be used to “evidences show” or “evidence show”.

Thank you very much for the positive assessment of the discussion. We have made the change in Line 110 to “evidence show.”

**Methods**

The method adopted for the study is apt and discussed in detail. Meanwhile, I do not have the expertise to comment on the model adopted. It is my hope that other reviewers will provide relevant feedback in that regard. However, most of the analyses are clear.

**Discussion**

The discussion section is well presented citing related previous studies

**Conclusion**

The conclusion section is concise but it is necessary to give more actionable recommendation talking about who does what.

Thank you, we have added state agencies and the government as actors who need to enact out the specific recommendations:

Lines x-y: “Reforms in scheme design which mandate gender-sensitive outreach by state agencies, ensure automatic or streamlined verification for all household members, and strengthen local health system capacity are needed in order to expand coverage. Government investments in district-level health infrastructure, administrative capacity, and community-based awareness campaigns can be useful in closing verification gaps.”

**Decision:** Accept with minor revision subject to Editor’s satisfaction.

**Date:** 14/04/2026

Regards,

**Afeez Lawal, Ph.D.**

**Response to Reviewer 2**

The manuscript titled *“Breaking down barriers to enrolment in health insurance: a multistate quantitative analysis of entitlement-based verification patterns under India’s PM-JAY Scheme”* addresses a highly relevant issue in both national and global health policy contexts. The focus on entitlement-based verification under PM‑JAY is timely and potentially impactful not only for India but other countries also. However, I have some observations and suggestions that may add value to the manuscript:

1. Clarity of Objective in the Abstract
   The authors aim to investigate the determinants of undergoing verification among PM‑JAY’s target population in order to identify underserved groups and inform strategies to expand coverage. This central objective should be explicitly and clearly stated in the abstract to help readers quickly grasp the study’s purpose.

We thank the reviewer for this suggestion. We have added the following text in line 43 to better reflect this:

“The objective of this study is to investigate determinants of undergoing verification among PM-JAY’s target population to identify underserved groups and inform strategies to expand coverage.”

2. Presentation of Key Quantitative Findings in the Abstract
   As this is a quantitative study, the abstract should report key statistical findings. This may include enrolment or verification status proportions, major associated factors, and statistically significant predictors of enrolment/verification. Presenting these results will enhance the informativeness and completeness of the abstract.

Thank you for this suggestion. We initially did not add the statistics to keep the text concise. We have now added the following statistical findings in the abstract (lines 53-58):

“Verification was more likely among older adults, with those ≥60years having higher odds than those 0-15 years of age (adjusted Odd Ratio [AOR] 2.339; 95% CI 2.071-2.641, p-value<0.001). Those with higher education had higher odds than those with no formal education (AOR 2.303; 95% CI 2.048-2.590, p-value<0.001), those with prior PFHI enrolment had higher odds compared to those not enrolled (AOR 1.947; 95% CI 1.827-2.075, p-value<0.001), and those with chronic illness had higher odds than those with no chronic conditions (AOR 1.175; 95% CI 1.071-1.289, p-value<0.001).”

3. Historical Context in the Introduction
   The introduction would benefit from a brief overview of the historical development of health insurance in India. This will provide important context for the emergence of PM‑JAY and situate the study within the broader evolution of India’s health financing landscape.

We have added the following historical context in lines 137-145:

“PM-JAY was formed by consolidating the previous national PHFI, the national Rashtriya Swasthya Bima Yojana (RSBY) scheme and other, state-level PFHI schemes. Historically, many Indian states started launching PFHI schemes after the passage of the Insurance Regulatory and Development Authority of India Act in 1999 which enabled health insurance reform and entry of private insurance providers; these schemes covered inpatient services and included the 2007 Rajiv Aarogyasri Scheme (in Andhra Pradesh), the 2008 central RSBY, and the 2009 Vajpayee Arogyasree Scheme, among others (12). RSBY followed these schemes, and was the largest such scheme for informal workers and the marginalized sections of society, but suffered from cumbersome enrolment procedures (13). Under all of these schemes and under RSBY,…”

4. Strengthening the Methods Section
   The Methods section requires additional detail and standardization:

- A brief description of the research instrument/tool used, including its development and validation process, should be added.

Thank you, we have added the following text in lines 200-216, under new headings as suggested in the next comment:

“**Tool development and validation:** The survey questionnaire captured household characteristics, socioeconomic background, and PM-JAY-specific information, including whether members had undergone verification and their experience of this process. A survey module on household consumption expenditure as a proxy for household wealth was developed, using a 30-day recall period for more frequently consumed items and a 365-day recall period for large purchases. Survey questions were developed using standard questions used in other validated surveys, such as the Indian government’s Survey on Household Social Consumption: Health (18), the Household Consumption Expenditure Survey (18) and the Demographic and Health Surveys. Survey questions on PM-JAY-specific information were developed using PM-JAY scheme beneficiary process guidelines (19). The questionnaire was translated into six regional languages. The translated questionnaire was piloted in peri-urban areas of Ahmedabad (Gujarat state) in June 2019, in Ghaziabad (Uttar Pradesh state) in July 2019, and in Nadia (West Bengal state) in August 2019. The piloting exercise was used to test the applicability, flow, consistency and language of questions, skip patterns, and estimate the length of interviews. Special care was taken to pilot questions on consumption expenditure of the household and their recall periods. Data were collected by locally recruited interviewer teams from the study districts who were fluent in the local language, using computer-assisted personal interviewing (CAPI) on digital devices. More detailed information is available in the study protocol (20).”

- Please align the reporting more closely with STROBE guidelines for observational studies, including:
  - Sample size calculation and rationale
  - Sampling method and procedures
  - Inclusion and exclusion criteria
  - Tools Development and its validation processes
  - Respondent selection process
  - Quality assurance measures

We have added this information under the suggested headings, as also specified in the response to the previous comment in lines 182-227 in the amended text:

“**Sampling method and procedures:** We used a subset of household survey data collected for a demand- and supply-side evaluation of the PM-JAY (17). Data were collected from PM-JAY-eligible households in 5 Indian States (Bihar, Chhattisgarh, Gujarat, Meghalaya, and Uttar Pradesh) during December 2019-February 2020; this period constituted the early implementation stage of PM-JAY. The states were selected in consultation with policy stakeholders to ensure geographic diversity and to represent different models of PM-JAY implementation. In this paper, we did not use data from two states in the original study (Kerala and Tamil Nadu) because they have different verification procedures compared to national guidelines.

Within each state, two districts (three in Bihar and Uttar Pradesh) classified as well- or average-performing in PM-JAY implementation were chosen; low-performing districts were excluded due to incomplete scheme implementation and expected poor outcomes. In each district, the primary sampling units (PSUs) comprised villages in rural or peri-urban areas (<7,500 inhabitants). Fifty PSUs were randomly selected to be representative of district-level PM-JAY characteristics, including the number of eligible households, number of claims, and broader demographic features (e.g., age, sex, marital status, and household composition) (39). From each PSU, 15 households were randomly selected. This entailed a sample of approximately 1150 households per district. Further details of the sampling strategy are provided in De Allegri et al. (2020) (17). The districts have been anonymized for analysis.

**Tool development and validation:** The survey questionnaire captured household characteristics, socioeconomic background, and PM-JAY-specific information, including whether members had undergone verification and their experience of this process. A survey module on household consumption expenditure as a proxy for household wealth was developed, using a 30-day recall period for more frequently consumed items and a 365-day recall period for large purchases. Survey questions were developed using standard questions used in other validated surveys, such as the Indian government’s Survey on Household Social Consumption: Health (18), the Household Consumption Expenditure Survey (18) and the Demographic and Health Surveys. Survey questions on PM-JAY-specific information were developed using PM-JAY scheme beneficiary process guidelines (19). The questionnaire was translated into six regional languages. The translated questionnaire was piloted in peri-urban areas of Ahmedabad (Gujarat state) in June 2019, in Ghaziabad (Uttar Pradesh state) in July 2019, and in Nadia (West Bengal state) in August 2019. The piloting exercise was used to test the applicability, flow, consistency and language of questions, skip patterns, and estimate the length of interviews. Special care was taken to pilot questions on consumption expenditure of the household and their recall periods. Data were collected by locally recruited interviewer teams from the study districts who were fluent in the local language, using computer-assisted personal interviewing (CAPI) on digital devices. More detailed information is available in the study protocol (20).

**Respondent selection process:** The head of household, or another knowledgeable adult in the sampled household served as the questionnaire respondent.

**Quality assurance measures:** Quality assurance measures before data collection included use of validated questions, piloting of the questionnaire, development of a survey field manual, and two week-long training of interviewers. During data collection, built-in electronic checks in the CAPI, monitoring of data collection and daily debrief sessions. After data collection, detailed data dictionaries and documentation of cleaning decisions were used to maintain data quality.

The final dataset included 9,833 households (50,002 individuals). Of these, 2,276 household heads (or another adult member when the head was unavailable) who reported undergoing the verification process were asked detailed questions about their verification experiences. We received all data as fully anonymized, secondary data.”

- Clearly explain the rationale for the chosen age-group categorization.

We have added the following rationale in lines 242-245:

“…(grouped into categories reflecting key life-course and labour-market stages [0–15, 16–29, 30–39, 40–49, 50–59, and 60+ years], consistent with commonly used demographic age classifications and with the distinction between dependent, prime working-age, and older adult populations)…”

- Provide a precise description of how household consumption (HCQ) was measured and operationalized.

The measurement and operationalization of household consumption expenditure was based on the exact method used in the Indian national Household Social Consumption Surveys. We have added the following explanation in lines 233-241 to reflect this:

“Household consumption expenditure was estimated from detailed item-wise reports of the quantity and value of goods and services consumed over specified reference periods (for example, the last 30, or 365 days, depending on the item group). For each household, expenditures across all reported items were summed to obtain total household consumption expenditure over the reference period, which was then converted to a monthly value and divided by household size to derive monthly per capita consumption expenditure (MPCE). Within each state, households were divided into quintiles of MPCE to account for state-level variations in consumption and wealth. This estimation followed the exact method and item lists used in the Indian national Household Social Consumption Surveys (18).”

5. Philosophical and Theoretical Linkage
   Incorporating a philosophical or theoretical framework (such as social justice, health equity theories) would strengthen the analytical depth of the study and better justify the interpretation of findings.

We thank the reviewer for highlighting the importance of social justice and health equity perspectives. We agree that PM-JAY is part of India’s broader commitment to universal health coverage and to “leaving no one behind”, and we now make this explicit in the introduction and discussion. However, the present manuscript is designed as an empirical assessment of verification patterns among eligible beneficiaries to inform program outreach and coverage improvement. Our data and analytic approach are not suited to formally operationalize or test a specific philosophical framework (e.g. theories of justice or capabilities), and adding such a framework would not alter the empirical analysis or strengthen its internal validity. We therefore opted to incorporate a concise equity and UHC framing, while keeping the conceptual scope aligned with the paper’s empirical and operational focus. We have added the following lines to better reflect this:

Lines 155-158: “PM-JAY is therefore a central pillar of India’s progress towards UHC, with an explicit focus on extending financial protection and access to hospital care to socioeconomically vulnerable households, consistent with the Sustainable Development Goal commitment to “leave no one behind” (15).”

Lines 352-356: “Viewed through a universal health coverage lens, these verification gaps are important because they limit effective access to a publicly financed entitlement intended to reduce financial barriers for vulnerable households. The persistence of such gaps suggests that formal eligibility alone is insufficient to ensure equitable coverage.”

6. Policy Context
   The manuscript should explicitly reference relevant national policies related to PM‑JAY, health insurance, and Universal Health Coverage (UHC). This will enhance policy relevance and clarify how the findings contribute to ongoing health system reforms in India.

Thank you. We have added references to the following national policies in the discussion in the relevant paragraphs:

Lines 428-432: “The Indian National Health Policy 2017 (43) outlines a roadmap for addressing specific gaps in health infrastructure and service capacity, while the Ayushman Bharat Digital Mission 2021 (44)promotes integrated and interoperable digital systems to improve service delivery; both initiatives can be aligned with PM-JAY processes to maximize equitable reach.”

Lines 436-439: “The Indian national AADHAAR biometric ID system (43), which is used across the country as a national identifier, can be one possible solution to replace the numerous documentation requirements; this may simplify identification and enable easier utilization of benefits across states.”

7. Alignment Between Title and Content
   Upon reviewing the full text, there appears to be a partial mismatch between the article title and the content presented. The authors are encouraged to reconsider whether the title accurately reflects the scope, focus, and analyses described in the manuscript, and to revise it if necessary for better alignment.

Thank you for this suggestion. We have changed the title to the following, to better reflect the article content about verification gaps:

Bridging the gap between health insurance eligibility and uptake: a multistate quantitative analysis of entitlement-based verification patterns under India’s PM-JAY Scheme
