# Supplementary material for: Bridging the gap between health insurance eligibility and uptake: A multistate quantitative analysis of entitlement-based verification patterns under India’s PM-JAY Scheme
Source: PLOS Glob Public Health. 2026 Oct 13;6(10):e0007451. doi: 10.1371/journal.pgph.0007451 (PMC13660894; doi:10.1371/journal.pgph.0007451)
Supplement: Supplementary file 10 [file pgph.0007451.s006.docx]

July 17, 2026

Dear Dr Kokkinias and Reviewers,

We thank you for the thoughtful reviews of our manuscript and your encouraging remarks on the revisions made. We have carefully considered the additional comments and have expanded the conclusion and framing of the study with the existing literature on the subject. We feel that the manuscript has greatly improved as a result of this. We have also conducted a thorough check of language usage, spelling, and grammar.

Below we provide a point-by-point response, and the corresponding edits which have been made in the manuscript.

We welcome any further suggestions you may have.

Kind regards,

All authors

**Response to Reviewer 1**

We thank the reviewer for their careful review and positive assessment of the manuscript.

**Response to Reviewer 2**

*The manuscript has improved substantially compared to the previous version. I sincerely appreciate the authors for their commendable efforts in revising the paper and addressing the earlier comments. To further strengthen the quality of the manuscript, I recommend the following revisions:*

*1. Include findings from studies conducted in neighboring countries to facilitate a comparative discussion of the results, highlighting similarities and differences where appropriate.*

Thank you for this feedback. We have referenced evidence from Nepal (lines 421-423) and from Pakistan (lines 361-364; lines 421-423), where large publicly-funded health insurance schemes exist. While the Nepali scheme includes an enrolment procedure with subsidized premiums, Pakistan’s Sehat Sahulat scheme has been made universal in select provinces, with similar gaps in attaining effective universal coverage. Bangladesh does not have a similar publicly-funded national health insurance scheme.

Lines 361-364:

In neighbouring Pakistan, the province of Khyber Pakhtunkhwa has expanded the national Sehat Sahulat inpatient PFHI to universal coverage; however, entitlement has not translated into effective universal coverage, with similar challenges around beneficiary enrolment (23,24).

Lines 421-423:

Closer in the region, infrastructural capacity differences across provinces and inadequate assistance for enrolment procedures have hindered enrolment in the Nepal Health Insurance Program (45–47) and in Pakistan’s Sehat Sahulat scheme (24).

*2. Ensure that the manuscript fully complies with the journal's formatting and submission guidelines.*

We have prepared the manuscript in accordance with the journal submission guidelines available here: <https://journals.plos.org/globalpublichealth/s/submission-guidelines>

*3. Carefully verify all numbers, percentages, and data presentations for accuracy and consistency. In addition, I recommend that the manuscript be thoroughly reviewed by a professional English language editor to improve clarity and readability.*

We have conducted a thorough check of all numbers and data, and performed a check of language use, spelling and grammar. We have made some minor edits through this check to improve consistency and readability.

*4. Expand the conclusion section by including recommendations for future research based on the study findings.*

Thank you for this feedback. Based on the study findings, we have included the following recommendations in lines 482-487:

Future research should systematically investigate the motivations and barriers that shape decisions to take up health insurance, with particular attention to population groups currently left out of coverage, such as the poor, less educated populations and women. Building on these insights, studies should employ qualitative and participatory approaches to co-develop and test targeted strategies that effectively reach, engage, and retain these excluded groups in health insurance schemes.

*Thank you very much for your dedication and the considerable effort invested in improving this manuscript. I wish the authors every success in the final revision.*

We thank the reviewer for the additional feedback and positive assessment of the manuscript.
