# Supplementary material for: Bridging the gap between health insurance eligibility and uptake: A multistate quantitative analysis of entitlement-based verification patterns under India’s PM-JAY Scheme
Source: PLOS Glob Public Health. 2026 Oct 13;6(10):e0007451. doi: 10.1371/journal.pgph.0007451 (PMC13660894; doi:10.1371/journal.pgph.0007451)
Supplement: Peer review history

# Peer review: Decision Letter 0

**DOI:** 10.1371/journal.pgph.0007451.r001

**Authors:** Katherine Kokkinias

26 Apr 2026

PGPH-D-26-00790

Breaking down barriers to enrolment in health insurance: a multistate quantitative analysis of entitlement-based verification patterns under India’s PM-JAY Scheme

PLOS Global Public Health

Dear Dr. Srivastava,

Thank you for submitting your manuscript to PLOS Global Public Health. After careful consideration, we feel that it has merit but does not fully meet PLOS Global Public Health’s publication criteria as it currently stands. Therefore, we invite you to submit a revised version of the manuscript that addresses the points raised during the review process.

The manuscript has been evaluated by two reviewers, and their comments are available below.

The reviewers have raised a number of concerns that need attention. They request additional information on methodological aspects of the study (such as a sample size calculation and inclusion criteria for households), description of how household consumption was measure and operationalized and further discussion of how these finding contribute to ongoing health system reforms in India.

Could you please revise the manuscript to carefully address the concerns raised?

Please submit your revised manuscript by Jun 23 2026 11:59PM. If you will need more time than this to complete your revisions, please reply to this message or contact the journal office at globalpubhealth@plos.org. When you're ready to submit your revision, log on to https://www.editorialmanager.com/pgph/ and select the 'Submissions Needing Revision' folder to locate your manuscript file.

Please include the following items when submitting your revised manuscript:

- A letter that responds to each point raised by the editor and reviewer(s). You should upload this letter as a separate file labeled 'Response to Reviewers'.
- A marked-up copy of your manuscript that highlights changes made to the original version. You should upload this as a separate file labeled 'Revised Manuscript with Track Changes'.
- An unmarked version of your revised paper without tracked changes. You should upload this as a separate file labeled 'Manuscript'.

Guidelines for resubmitting your figure files are available below the reviewer comments at the end of this letter.

As the corresponding author, your ORCID iD is verified in the submission system and will appear in the published article. PLOS supports the use of ORCID, and we encourage all coauthors to register for an ORCID iD and use it as well. Please encourage your coauthors to verify their ORCID iD within the submission system before final acceptance, as unverified ORCID iDs will not appear in the published article. *Only*  the individual author can complete the verification step; PLOS staff *cannot*  verify ORCID iDs on behalf of authors.

We look forward to receiving your revised manuscript.

Kind regards,

Katherine Demi Kokkinias, Ph.D.

Staff Editor

PLOS Global Public Health

Journal Requirements:

1. Please include a complete copy of PLOS’ questionnaire on inclusivity in global research in your revised manuscript. Our policy for research in this area aims to improve transparency in the reporting of research performed outside of researchers’ own country or community. The policy applies to researchers who have travelled to a different country to conduct research, research with Indigenous populations or their lands, and research on cultural artefacts. The questionnaire can also be requested at the journal’s discretion for any other submissions, even if these conditions are not met. Please find more information on the policy and a link to download a blank copy of the questionnaire here: https://journals.plos.org/globalpublichealth/s/best-practices-in-research-reporting. Please upload a completed version of your questionnaire as Supporting Information when you resubmit your manuscript.

If the reviewer comments include a recommendation to cite specific previously published works, please review and evaluate these publications to determine whether they are relevant and should be cited. There is no requirement to cite these works unless the editor has indicated otherwise.

Additional Editor Comments (if provided):

[Note: HTML markup is below. Please do not edit.]

Reviewers' comments:

Reviewer's Responses to Questions

**Comments to the Author**

1. Does this manuscript meet PLOS Global Public Health’s publication criteria?

Reviewer #1: Yes

Reviewer #2: Partly

**********

2. Has the statistical analysis been performed appropriately and rigorously?

Reviewer #1: I don't know

Reviewer #2: I don't know

**********

3. Have the authors made all data underlying the findings in their manuscript fully available (please refer to the Data Availability Statement at the start of the manuscript PDF file)?

The PLOS Data policy

Reviewer #1: Yes

Reviewer #2: No

**********

4. Is the manuscript presented in an intelligible fashion and written in standard English?

Reviewer #1: Yes

Reviewer #2: Yes

**********

Reviewer #1: Background

The authors gave an insight to the issues of health insurance enrolment in the PM-JAY scheme in India. The discussion is well presented as it gave some examples from the LMICs. However, on Line 110, plural form of evidence should be used to “evidences show” or “evidence show”.

Methods

The method adopted for the study is apt and discussed in detail. Meanwhile, I do not have the expertise to comment on the model adopted. It is my hope that other reviewers will provide relevant feedback in that regard. However, most of the analyses are clear.

Discussion

The discussion section is well presented citing related previous studies.

Conclusion

The conclusion section is concise but it is necessary to give more actionable recommendation talking about who does what.

Decision: Accept with minor revision subject to Editor’s satisfaction.

Reviewer #2: The manuscript titled “Breaking down barriers to enrolment in health insurance: a multistate quantitative analysis of entitlement-based verification patterns under India’s PM-JAY Scheme” addresses a highly relevant issue in both national and global health policy contexts. The focus on entitlement-based verification under PM JAY is timely and potentially impactful not only for India but other countries also. However, I have some observations and suggestions that may add value to the manuscript:

1. Clarity of Objective in the Abstract

The authors aim to investigate the determinants of undergoing verification among PM JAY’s target population in order to identify underserved groups and inform strategies to expand coverage. This central objective should be explicitly and clearly stated in the abstract to help readers quickly grasp the study’s purpose.

2. Presentation of Key Quantitative Findings in the Abstract

As this is a quantitative study, the abstract should report key statistical findings. This may include enrolment or verification status proportions, major associated factors, and statistically significant predictors of enrolment/verification. Presenting these results will enhance the informativeness and completeness of the abstract.

3. Historical Context in the Introduction

The introduction would benefit from a brief overview of the historical development of health insurance in India. This will provide important context for the emergence of PM JAY and situate the study within the broader evolution of India’s health financing landscape.

4. Strengthening the Methods Section

The Methods section requires additional detail and standardization:

� A brief description of the research instrument/tool used, including its development and validation process, should be added.

� Please align the reporting more closely with STROBE guidelines for observational studies, including:

� Sample size calculation and rationale

� Sampling method and procedures

� Inclusion and exclusion criteria

� Tools Development and its validation processes

� Respondent selection process

� Quality assurance measures

� Clearly explain the rationale for the chosen age-group categorization.

� Provide a precise description of how household consumption (HCQ) was measured and operationalized.

5. Philosophical and Theoretical Linkage

Incorporating a philosophical or theoretical framework (such as social justice, health equity theories) would strengthen the analytical depth of the study and better justify the interpretation of findings.

6. Policy Context

The manuscript should explicitly reference relevant national policies related to PM JAY, health insurance, and Universal Health Coverage (UHC). This will enhance policy relevance and clarify how the findings contribute to ongoing health system reforms in India.

7. Alignment Between Title and Content

Upon reviewing the full text, there appears to be a partial mismatch between the article title and the content presented. The authors are encouraged to reconsider whether the title accurately reflects the scope, focus, and analyses described in the manuscript, and to revise it if necessary for better alignment.

**********

what does this mean?). If published, this will include your full peer review and any attached files.

**Do you want your identity to be public for this peer review?**  If you choose “no”, your identity will remain anonymous but your review may still be made public.

For information about this choice, including consent withdrawal, please see our Privacy Policy

Reviewer #1: No

Reviewer #2: No

**********

[NOTE: If reviewer comments were submitted as an attachment file, they will be attached to this email and accessible via the submission site. Please log into your account, locate the manuscript record, and check for the action link "View Attachments". If this link does not appear, there are no attachment files.]

Attachment Submitted filename: Paper Review Breaking down barriers.docx

Attachment Submitted filename: Comment.docx

# Author comment: Author response to Decision Letter 1

**DOI:** 10.1371/journal.pgph.0007451.r002

23 Jun 2026

Attachment Submitted filename: Response Letter_2026.06.09.docx

# Peer review: Decision Letter 1

**DOI:** 10.1371/journal.pgph.0007451.r003

**Authors:** Katherine Kokkinias

16 Jul 2026

PGPH-D-26-00790R1

Bridging the gap between health insurance eligibility and uptake: a multistate quantitative analysis of entitlement-based verification patterns under India’s PM-JAY Scheme

PLOS Global Public Health

Dear Dr. Srivastava,

Thank you for submitting your manuscript to PLOS Global Public Health. After careful consideration, we feel that it has merit but does not fully meet PLOS Global Public Health’s publication criteria as it currently stands. Therefore, we invite you to submit a revised version of the manuscript that addresses the points raised during the review process.

The manuscript has been evaluated by two reviewers, and their comments are available below.

The reviewers have raised additional concerns that need attention. They requested expansion of the conclusion and framing of the study within the existing literature on the subject.

In addition, a reviewer has suggested that you thoroughly copyedit your manuscript for language usage, spelling, and grammar. If you do not know anyone who can help you do this, you may wish to consider employing a professional scientific editing service.

Whilst you may use any professional scientific editing service of your choice, PLOS has partnered with both American Journal Experts (AJE) and Editage to provide discounted services to PLOS authors. Both organizations have experience helping authors meet PLOS guidelines and can provide language editing, translation, manuscript formatting, and figure formatting to ensure your manuscript meets our submission guidelines. To take advantage of our partnership with AJE, visit the AJE website ( http://learn.aje.com/plos/) for a 15% discount off AJE services. To take advantage of our partnership with Editage, visit the Editage website (www.editage.com) and enter referral code PLOSEDIT for a 15% discount off Editage services. If the PLOS editorial team finds any language issues in text that either AJE or Editage has edited, the service provider will re-edit the text for free.

Upon resubmission, please provide the following:

- The name of the colleague or the details of the professional service that edited your manuscript
- A copy of your manuscript showing your changes by either highlighting them or using track changes (uploaded as a *supporting information* file)
- A clean copy of the edited manuscript (uploaded as the new *manuscript* file)

Could you please revise the manuscript to carefully address the concerns raised?

Please submit your revised manuscript by Sep 14 2026 11:59PM. If you will need more time than this to complete your revisions, please reply to this message or contact the journal office at globalpubhealth@plos.org. When you're ready to submit your revision, log on to https://www.editorialmanager.com/pgph/ and select the 'Submissions Needing Revision' folder to locate your manuscript file.

Please include the following items when submitting your revised manuscript:

- A letter that responds to each point raised by the editor and reviewer(s). You should upload this letter as a separate file labeled 'Response to Reviewers'.
- A marked-up copy of your manuscript that highlights changes made to the original version. You should upload this as a separate file labeled 'Revised Manuscript with Track Changes'.
- An unmarked version of your revised paper without tracked changes. You should upload this as a separate file labeled 'Manuscript'.

Guidelines for resubmitting your figure files are available below the reviewer comments at the end of this letter.

As the corresponding author, your ORCID iD is verified in the submission system and will appear in the published article. PLOS supports the use of ORCID, and we encourage all coauthors to register for an ORCID iD and use it as well. Please encourage your coauthors to verify their ORCID iD within the submission system before final acceptance, as unverified ORCID iDs will not appear in the published article. *Only*  the individual author can complete the verification step; PLOS staff *cannot*  verify ORCID iDs on behalf of authors.

We look forward to receiving your revised manuscript.

Kind regards,

Katherine Demi Kokkinias, Ph.D.

Staff Editor

PLOS Global Public Health

Journal Requirements:

If the reviewer comments include a recommendation to cite specific previously published works, please review and evaluate these publications to determine whether they are relevant and should be cited. There is no requirement to cite these works unless the editor has indicated otherwise.

Additional Editor Comments (if provided):

[Note: HTML markup is below. Please do not edit.]

Reviewers' comments:

Reviewer's Responses to Questions

**Comments to the Author**

Reviewer #1: All comments have been addressed

Reviewer #2: All comments have been addressed

**********

publication criteria?

Reviewer #1: Yes

Reviewer #2: Partly

**********

3. Has the statistical analysis been performed appropriately and rigorously?

Reviewer #1: I don't know

Reviewer #2: I don't know

**********

4. Have the authors made all data underlying the findings in their manuscript fully available (please refer to the Data Availability Statement at the start of the manuscript PDF file)?

The PLOS Data policy

Reviewer #1: Yes

Reviewer #2: Yes

**********

5. Is the manuscript presented in an intelligible fashion and written in standard English?

Reviewer #1: Yes

Reviewer #2: Yes

**********

**Review Comments to the Author**

Reviewer #1: No comments.

Reviewer #2: The manuscript has improved substantially compared to the previous version. I sincerely appreciate the authors for their commendable efforts in revising the paper and addressing the earlier comments. To further strengthen the quality of the manuscript, I recommend the following revisions:

1. Include findings from studies conducted in neighboring countries to facilitate a comparative discussion of the results, highlighting similarities and differences where appropriate.

2. Ensure that the manuscript fully complies with the journal's formatting and submission guidelines.

3. Carefully verify all numbers, percentages, and data presentations for accuracy and consistency. In addition, I recommend that the manuscript be thoroughly reviewed by a professional English language editor to improve clarity and readability.

4. Expand the conclusion section by including recommendations for future research based on the study findings.

Thank you very much for your dedication and the considerable effort invested in improving this manuscript. I wish the authors every success in the final revision.

**********

what does this mean?). If published, this will include your full peer review and any attached files.

**Do you want your identity to be public for this peer review?**  If you choose “no”, your identity will remain anonymous but your review may still be made public.

For information about this choice, including consent withdrawal, please see our Privacy Policy

Reviewer #1: No

Reviewer #2: **Yes:**  Devaraj Acharya, PhD

**********

[NOTE: If reviewer comments were submitted as an attachment file, they will be attached to this email and accessible via the submission site. Please log into your account, locate the manuscript record, and check for the action link "View Attachments". If this link does not appear, there are no attachment files.]

# Author comment: Author response to Decision Letter 2

**DOI:** 10.1371/journal.pgph.0007451.r004

20 Jul 2026

Attachment Submitted filename: Response Letter_2026.07.17.docx

# Peer review: Decision Letter 2

**DOI:** 10.1371/journal.pgph.0007451.r005

**Authors:** Alejandro Torrado Pacheco

12 Aug 2026

PGPH-D-26-00790R2

Bridging the gap between health insurance eligibility and uptake: a multistate quantitative analysis of entitlement-based verification patterns under India’s PM-JAY Scheme

PLOS Global Public Health

Dear Dr. Srivastava,

Thank you for submitting your manuscript to PLOS Global Public Health. After careful consideration, we feel that it has merit but does not fully meet PLOS Global Public Health’s publication criteria as it currently stands. Therefore, we invite you to submit a revised version of the manuscript that addresses the points raised during the review process.

The manuscript has been evaluated by three reviewers, and their comments are available below. Reviewer 3 has highlighted several issues with the statistical analyses that need to be addressed. Could you please revise the manuscript to carefully address the concerns raised?

Please submit your revised manuscript by Oct 10 2026 11:59PM. If you will need more time than this to complete your revisions, please reply to this message or contact the journal office at globalpubhealth@plos.org. When you're ready to submit your revision, log on to https://www.editorialmanager.com/pgph/ and select the 'Submissions Needing Revision' folder to locate your manuscript file.

Please include the following items when submitting your revised manuscript:

- A letter that responds to each point raised by the editor and reviewer(s). You should upload this letter as a separate file labeled 'Response to Reviewers'.
- A marked-up copy of your manuscript that highlights changes made to the original version. You should upload this as a separate file labeled 'Revised Manuscript with Track Changes'.
- An unmarked version of your revised paper without tracked changes. You should upload this as a separate file labeled 'Manuscript'.

Guidelines for resubmitting your figure files are available below the reviewer comments at the end of this letter.

As the corresponding author, your ORCID iD is verified in the submission system and will appear in the published article. PLOS supports the use of ORCID, and we encourage all coauthors to register for an ORCID iD and use it as well. Please encourage your coauthors to verify their ORCID iD within the submission system before final acceptance, as unverified ORCID iDs will not appear in the published article. *Only*  the individual author can complete the verification step; PLOS staff *cannot*  verify ORCID iDs on behalf of authors.

We look forward to receiving your revised manuscript.

Kind regards,

Alejandro Torrado Pacheco, PhD

PLOS Editor

PLOS Global Public Health

**Journal Requirements:**

If the reviewer comments include a recommendation to cite specific previously published works, please review and evaluate these publications to determine whether they are relevant and should be cited. There is no requirement to cite these works unless the editor has indicated otherwise.

**Additional Editor Comments (if provided):**

[Note: HTML markup is below. Please do not edit.]

Reviewers' comments:

Reviewer's Responses to Questions

**Comments to the Author**

Reviewer #1: All comments have been addressed

Reviewer #2: All comments have been addressed

Reviewer #3: (No Response)

**********

publication criteria?

Reviewer #1: Yes

Reviewer #2: Partly

Reviewer #3: (No Response)

**********

3. Has the statistical analysis been performed appropriately and rigorously?

Reviewer #1: I don't know

Reviewer #2: Yes

Reviewer #3: Yes

**********

4. Have the authors made all data underlying the findings in their manuscript fully available (please refer to the Data Availability Statement at the start of the manuscript PDF file)?

The PLOS Data policy

Reviewer #1: Yes

Reviewer #2: No

Reviewer #3: Yes

**********

5. Is the manuscript presented in an intelligible fashion and written in standard English?

Reviewer #1: Yes

Reviewer #2: Yes

Reviewer #3: Yes

**********

**Reviewer #1** : Satisfactory

**Reviewer #2:**  Great, congratulation!

**Reviewer #3:**  As the statistical reviewer I will focus on methods and reporting. Note that this is the first time I see this paper.

Major

1) The manuscript should describe how missing data were handled in the analysis. At present, it is not clear whether a complete-case analysis was used, whether any observations were excluded because of missing covariates, or what the level of missingness was for each variable. The authors should report missingness clearly, ideally by variable, and consider a sensitivity analysis using multiple imputation if missingness is non-trivial.

2) The modelling approach is broadly appropriate for a binary outcome, and the use of district-level random effects is reasonable given likely differences in implementation across districts. However, the analysis is conducted at the individual level while individuals are nested within households, so the authors should clarify whether household-level clustering was accounted for, and if not, consider this or justify its omission. The model should also more clearly state which covariates were included and why. The bivariate analyses appear mainly descriptive, the authors should clarify their purpose and confirm that univariable p-values were not used for model selection

3) The results place considerable emphasis on statistical significance, including repeated use of p-values. As the sample size is large, small differences may become statistically significant without necessarily being substantively important. The authors should place greater emphasis on the size and direction of associations, and the uncertainty around estimates, and interpret the findings in terms of practical or policy relevance rather than statistical significance alone.

Minor

1) The manuscript does not state whether reporting followed an appropriate checklist, such as STROBE for observational studies, and I could not see a completed checklist among the submitted materials.

**********

what does this mean?). If published, this will include your full peer review and any attached files.

**Do you want your identity to be public for this peer review?**  If you choose “no”, your identity will remain anonymous but your review may still be made public.

For information about this choice, including consent withdrawal, please see our Privacy Policy

Reviewer #1: **Yes:**  Afeez Folorunsho Lawal

Reviewer #2: No

Reviewer #3: No

**********

[NOTE: If reviewer comments were submitted as an attachment file, they will be attached to this email and accessible via the submission site. Please log into your account, locate the manuscript record, and check for the action link "View Attachments". If this link does not appear, there are no attachment files.]

**Figure Resubmissions:**

# Author comment: Author response to Decision Letter 3

**DOI:** 10.1371/journal.pgph.0007451.r006

21 Sep 2026

Attachment Submitted filename: Response Letter_2026.09.21.docx

# Peer review: Decision Letter 3

**DOI:** 10.1371/journal.pgph.0007451.r007

**Authors:** Anil Fastenau

23 Sep 2026

Bridging the gap between health insurance eligibility and uptake: a multistate quantitative analysis of entitlement-based verification patterns under India’s PM-JAY Scheme

PGPH-D-26-00790R3

Dear Dr Srivastava,

We are pleased to inform you that your manuscript 'Bridging the gap between health insurance eligibility and uptake: a multistate quantitative analysis of entitlement-based verification patterns under India’s PM-JAY Scheme' has been provisionally accepted for publication in PLOS Global Public Health.

Before your manuscript can be formally accepted you will need to complete some formatting changes, which you will receive in a follow up email. A member of our team will be in touch with a set of requests.

Please note that your manuscript will not be scheduled for publication until you have made the required changes, so a swift response is appreciated.

IMPORTANT: The editorial review process is now complete. PLOS will only permit corrections to spelling, formatting or significant scientific errors from this point onwards. Requests for major changes, or any which affect the scientific understanding of your work, will cause delays to the publication date of your manuscript.

If your institution or institutions have a press office, please notify them about your upcoming paper to help maximize its impact. If they'll be preparing press materials, please inform our press team as soon as possible -- no later than 48 hours after receiving the formal acceptance. Your manuscript will remain under strict press embargo until 2 pm Eastern Time on the date of publication. For more information, please contact globalpubhealth@plos.org.

Thank you again for supporting Open Access publishing; we are looking forward to publishing your work in PLOS Global Public Health.

Best regards,

Anil Fastenau, M.D., M.Sc.

Academic Editor

PLOS Global Public Health

***********************************************************

Reviewer Comments (if any, and for reference):

Reviewer's Responses to Questions

**Comments to the Author**

Reviewer #2: All comments have been addressed

Reviewer #3: All comments have been addressed

**********

publication criteria?

Reviewer #2: Partly

Reviewer #3: Yes

**********

3. Has the statistical analysis been performed appropriately and rigorously?

Reviewer #2: Yes

Reviewer #3: Yes

**********

4. Have the authors made all data underlying the findings in their manuscript fully available (please refer to the Data Availability Statement at the start of the manuscript PDF file)?

The PLOS Data policy

Reviewer #2: No

Reviewer #3: Yes

**********

5. Is the manuscript presented in an intelligible fashion and written in standard English?

Reviewer #2: Yes

Reviewer #3: Yes

**********

Reviewer #2: (No Response)

Reviewer #3: I am satisfied with the authors' responses and the resulting changes to the paper.

**********

what does this mean?). If published, this will include your full peer review and any attached files.

**Do you want your identity to be public for this peer review?**  If you choose “no”, your identity will remain anonymous but your review may still be made public.

For information about this choice, including consent withdrawal, please see our Privacy Policy

Reviewer #2: No

Reviewer #3: No

**********
